# High throughput screening for human disease associated-pathogens and antimicrobial resistance genes in migratory birds at ten habitat sites in China

**DOI:** 10.1186/s12866-025-04059-4

**Published:** 2025-06-06

**Authors:** Lan Wang, Ru Jia, Rufei Ma, Jie Li, Shanrui Wu, Yeshun Fan, Dan Zhao, Dianfeng Chu, Yihua Wang, Guogang Zhang, Jie Liu

**Affiliations:** 1 Department of Microbial Surveillance and Biosafety, School of Public Health, Qingdao University, Qingdao, Shandong China; 2 Key Laboratory of Biodiversity Conservation of National Forestry and Grassland Administration, Ecology and Nature Conservation Institute, Chinese Academy of Forestry, Beijing, China; 3 Yebio BioEngineering Co. Ltd of Qingdao, Qingdao, Shandong China

**Keywords:** Migratory birds, Pathogen, Antimicrobial resistance, TaqMan Array Card, Surveillance

## Abstract

**Background:**

Migratory birds have been found to carry and spread pathogens, contaminating the environment and causing diseases in humans and other animals. To our knowledge, there hasn’t been any systematic targeted screening for known pathogens in migratory birds. In the current study, customized real time PCR based TaqMan Array Cards (TAC) were used to detect 99 human disease related pathogens and 20 antimicrobial resistance (AMR) genes in migratory birds at 10 habitat sites in China.

**Results:**

The results showed that 30.5% (107/351) of migratory birds carried at least one of 14 pathogens. The most prevalent pathogens included *Aeromonas*,* Plasmodium*,* Cryptosporidium*,* Giardia lamblia*, enteropathogenic *Escherichia coli* (EPEC), *Campylobacter jejuni/coli*, and *Rickettsia*. Their distribution demonstrated certain host or region specificity. *Anseriformes* carried higher rate of pathogens (39.1%, 72/184) than *Charadriiformes* (23.2%, 33/142, *p* < 0.05). The overall pathogen detection rate was the highest in Hubei (87.1%, 27/31), possessing exclusively *Anser*. The pathogen quantities were estimated to be 10^3^ to 2 × 10^8^ gene copies per gram of feces. AMR genes associated with resistance to macrolides, quinolones, tetracyclines, and β-lactams were widely detected, with overall quantities ranging from 10^5^ to 10^9^ copies of interrogated genes for each drug class per gram of feces.

**Conclusions:**

Using such a multi-target detection and quantification platform, this study evaluated the potential role of migratory birds as reservoirs or vectors for a broad range of pathogens and AMR genes in the environment, indicating their capacity to transmit zoonotic diseases. These might provide evidence for implementation of targeted intervention with a one health approach.

**Supplementary Information:**

The online version contains supplementary material available at 10.1186/s12866-025-04059-4.

## Background

Migratory birds carry a variety of microorganisms during migration, expanding the geographical and host range of pathogen transmission, which may be involved in the epidemiological chain of human diseases through environmental pollution and other pathways, posing potential public health risks [1]. Since the first large-scale outbreak of highly pathogenic avian influenza at Qinghai Lake in China in 2005, people have reinforced the investigation of migratory birds [2, 3]. Human intrusion into the natural habitats of wild birds, domestication of wild birds as pets or racing birds, and increased human consumption of poultry may promote pathogen transmission, leading to the emergence and spread of zoonoses [4]. More than 1,400 human pathogens have been described, of which about 62% are classified as zoonotic. Emerging infectious disease events were dominated by zoonoses (60.3%), most of which (71.8%) originated in wild animals [5]. Meanwhile, migratory birds have been found to be the disseminators of antimicrobial resistance across human-bird-environment interfaces [6–8], particularly over long distance to pristine environment like Antarctica [9]. Therefore, systematic surveillance of pathogens and AMR genes carried by migratory birds has been highlighted with a view to identifying their potential transmission risks and hazards [10], which can be essential for assessing the animal sources of infection [11].

Among the nine global migratory routes of birds, four pass through China, covering majority of the territory. The surveillance of avian flu has been widely implemented, while some studies have focused on individual bacteria or viruses [12–16]. Recent metagenomic sequencing approach has often aimed to discover novel microorganisms. However, there is a lack of molecular surveillance and epidemiological studies targeting multiple regions, representative migratory birds along migratory routes for a variety of diseases causing microorganisms. Many pathogens that cause pneumonia, diarrhea, meningitis, and febrile illness in humans, can like be spread through the human-animal-environment interface [17–19].

Nucleic acid based molecular methods have become a powerful tool for targeted pathogen detection because of their higher efficiency and accuracy compared to conventional methods such as bacterial culture and ELISA [20]. In particular, multiplex PCR increases the throughput and simplifies the procedure. Additionally, quantitative PCR (qPCR) enables the quantification of the pathogens. TaqMan Array Card (TAC) is a multiplex qPCR based microfluidic card that harbors 48 individual qPCR reactions that are physically separated for each of the 8 samples. This approach is greatly beneficial when a large number of pathogens need to be screened, including human diseases and animal or environmental surveillance [21–26].

We previously developed TAC cards for the detection of respiratory, enteric, and bloodstream pathogens as well as AMR genes, and utilized them widely for the surveillance of the relevant human diseases [22, 27–31]. In the current study, we applied these TACs to screen for 99 human disease related pathogens and 20 AMR genes in feces of migratory birds collected from ten regions along the migration routes in China to assess their potential transmission through bird droppings in the environment.

## Methods

### Sample collection

A total of 351 fresh fecal dropping samples from migratory birds were collected at ten bird wintering/stopover sites in China from September 2019 to November 2023, including Xingkai Lake in Heilongjiang, Cangzhou in Hebei, Longbao Nature Reserve in Qinghai, Grand View Pavilion and Dianchi Lake in Yunnan, Minjiang estuary in Fujian, Lingwu City in Ningxia, Wanghu Lake in Hubei, Tumuji National Nature Reserve in Inner Mongolia, Qingdao Zhanqiao in Shandong, and counties along the Yarlung Zangbo River in Xizang. The samples were preserved in the transport medium (0.9% NaCl, 0.2 g/L penicillin, 2 g/L streptomycin sulfate, 20% glycerol), transported with cold chain, and stored at − 80℃ until testing.

### Total nucleic acid extraction

Nucleic acid extraction was performed using the cador Pathogen 96 QIAcube HT kit with the automated QIAcube HT system (Qiagen, Hilden, Germany). Briefly, 200 mg or 200 µL of fecal sample was mixed with 0.8 mL of InhibitEX buffer (Qiagen, Hilden, Germany) and glass beads of 212 ~ 300 μm (Sigma-Aldrich, St. Louis, USA). The mixture was homogenized for four times in the OMNI Bead Ruptor 24 Elite (Kennesaw GA, USA) at 8 m/s for 30 s with an interval of 20 s, followed by incubation at 95 °C for 5 min. 200 µL of supernatant were collected after centrifugation and loaded onto QIAcube HT system for automated nucleic acid extraction. Two external controls (Phocine herpesvirus for DNA targets and MS2 bacteriophage for RNA targets) were spiked into each sample to monitor extraction and amplification efficiency. Each batch of extraction included one extraction blank to rule out laboratory contamination.

### Detection of pathogens and AMR genes with TaqMan Array Cards

Three customized TaqMan Array Cards targeting pathogens (supplemental Table S1) and AMR genes (supplemental Table S2) involved in enteric, respiratory, and bloodstream infections, respectively, were adapted from previous studies [22, 27–31], and manufactured by Thermo Fisher (Carlsbad, CA, USA). All the qPCR assays have previously been extensively validated (supplemental Table S1). In order to improve the throughput, 5 samples were pooled with equal volume, i.e. 15 µL each, then mixed with 25 µL of TaqMan Fast Virus 1-Step Master Mix (4×) reagent (Thermo Fisher) and loaded into each port of the TAC card following the manufacturer’s instruction. Real time PCR was performed with QuantStudio7 Real-time PCR instrument (Thermo Fisher) and analyzed using QuantStudio7 Real-time PCR Software. The qPCR cycling conditions were set as 50℃ for 10 min, 95℃ for 20 s, 40 cycles of 95℃ for 3 s, and 60℃ for 30 s. Quantification cycle (Cq) of 35 was used to determine the positivity. The positive results were valid only when the corresponding extraction blank was negative for the relevant targets. The negative results were valid only when the corresponding external controls amplified with the expected signal. Pathogen and AMR gene copy numbers were further derived from Cq values based on the standard curves generated with pooled positive controls, and normalized as copy numbers per gram of feces.

### Confirmation of positive pathogen detections

When a pooled sample was positive for a certain pathogen, the 5 individual samples of the pool were tested with the cognate qPCR assay on 96-well plate to identify the positive sample. The 10-µL reaction contained 2.5 µL of TaqMan Fast Virus 1-Step Master Mix, 9 pmol of primers, 2.5 pmol of probe, and 2 µL of nucleic acid extract. The cycling condition was the same as those for TAC.

For samples that amplified for *Cryptosporidium* 18 S rRNA, qPCR targeting *COWP* gene and nested PCR for 18 S rRNA gene (supplemental Table S3) followed by amplicon sequencing (Tsingke, Qingdao, China) were performed to confirm *Cryptosporidium* detection [32, 33].

### Identification of bird types

For bird species identification, the published assay targeting mitochondrial cytochrome oxidase sub gene I (COI) were modified to accommodate a few more bird species in the current study [34], with the forward primer 5’- CACGAATAAACAACATAAGCTTCTG-3’, reverse primers 5’- CAGGGTGTCCGAAGAATC-3’ and 5’- CTGGGTGGCCRAARAATC-3’. The 20 µL reaction contained 10 µL of Taq Plus Master Mix II (Vazyme, Nanjing, China), 4 pmol of each primer, 1 µL of nucleic acid extract, and nuclease-free water up to 20 µL. The PCR cycling conditions were set as initial denaturation at 95 °C for 3 min, followed by 45 cycles of 95 °C for 15 s, 58 °C for 30 s, and 72 °C for 60 s, and a final extension at 72 °C for 10 min. The PCR products were examined with gel electrophoresis and sent for amplicon sequencing.

### Statistical analysis

The prevalence of multiple pathogens in each region was expressed as the percentage of samples positive by qPCR. One-sample t-tests were used to determine if there were statistical differences between regions and bird types. Pathogen quantities were compared with Mann-Whitney U test. SPSS software, version 26.0, was used for the analysis. Two-tailed *p* values were calculated, and values of < 0.05 were considered statistically significant.

## Results

### Distribution of migratory bird types

Based on our preliminary data with an average detection rate of 1.5% for major human disease associated pathogens such as *Campylobacter* and *Cryptosporidium* in migratory birds, the sample size per site was estimated to be 23. A total of 351 samples were collected as shown in Table 1. During fecal sample collection, the bird types were observed and recorded at some sites, including *Anser indicus* at Qinghai and Xizang sites, *Anser albifrons* at Heilongjiang and Inner Mongolia, and *Chroicocephalus ridibundus* at Yunnan and Shandong. These were confirmed by the COI amplicon sequencing (supplemental Table S4). Several *Anser* species were observed at Hubei site, while COI analysis identified them as *Anser cygnoides*, *Anser albifrons*, *Anser fabalis*, and *Anser anser*. A variety of migratory birds group lived at Fujian, Ningxia, and Hebei sites, and most were identified by COI as shown in Table 1. Overall, the bird species were identified for 94.3% (331/351) of the samples by COI sequencing, involving 15 genera in 4 orders (Table 1), with *Anseriformes* (52.4%, 184/351) and *Charadriiformes* (40.5%, 142/351) being the two predominate types.

### Pathogen detection by pooled TAC followed by individual confirmation

**Table 1:** Migratory bird types identified by COI PCR amplicon sequencing in this study

Bird Order	Anseriformes		Charadriiformes										Gruiformes		Lariformes	Other*	Total
Bird Genus	*Anser*	*Aythya*	*Calidris*	*Charadrius*	*Chlidonias*	*Chroicocephalus*	*Himantopus*	*Numenius*	*Philomachus*	*Pluvialis*	*Recurvirostra*	*Tringa*	*Fulica*	*Grus*	*Sterna*		
Fujian	16	-	8	7	-	1	-	5	-	-	-	-	-	-	-	6	43
Hebei	2	-	4	-	2	9	4	-	-	-	-	2	-	-	3	14	40
Heilongjiang	28	-	-	-	-	-	-	-	-	-	-	-	-	-	-	-	28
Hubei	31	-	-	-	-	-	-	-	-	-	-	-	-	-	-	-	31
Inner Mongolia	25	2	-	-	-	2	-	-	-	-	-	-	-	1	-	-	30
Ningxia	9	-	-	-	-	3	3	-	1	3	8	-	1	-	-	-	28
Qinghai	36	-	-	-	-	-	-	-	-	-	-	-	-	-	-	-	36
Shandong	-	-	-	-	-	28	-	-	-	-	-	-	-	-	-	-	28
Xizang	35	-	-	-	-	-	-	-	-	-	-	-	-	-	-	-	35
Yunnan	-	-	-	-	-	52	-	-	-	-	-	-	-	-	-	-	52
Total	184		142										2		3	20	351

* Bird species were not identified with COI

Through testing on pooled samples by TAC followed by confirmation on each individual samples with qPCR on plate, a total of 14 pathogens were detected positive, including 8 (8/49) bacteria, 1 (1/27) virus, and 5 (5/17) parasites (supplemental Figure S1). None of the 6 fungi interrogated was detected. Additionally, *Cryptosporidium* results was further confirmed by *COWP* qPCR and nested 18 S rRNA PCR followed by amplicon sequencing (supplemental Tables S3 and S4) because the *Cryptosporidium* qPCR assay on TAC may detect algae that are genetically close and commonly present in the wetland [35]. At least one pathogen was detected in 107 samples (30.5%) (Table 2). *Aeromonas* (9.1%), *Plasmodium* (7.4%), and *Cryptosporidium* (5.1%) were the three most prevalent pathogens (Table 2). HIV I (0.9%) was the only virus detected.

### Distribution of pathogens in migratory birds by region and bird type

At least one pathogen was detected at each of the 10 habitat sites interrogated in the current study (Table 2), with Hubei (87.1%, 27/31) and Ningxia (64.3%, 18/28) having the highest detection rates while Hebei (7.5%, 3/40) and Fujian (2.3%, 1/43) had the least (*p* < 0.05). From the perspective of geographical distribution, multiple pathogens were detected in multiple regions. *Cryptosporidium* had a wide geographic distribution across 7 of the 10 sites (Table 2). On the contrary, some pathogens showed specificity to a certain region or a certain bird species, such as *Rickettsia* in *Charadriiformes* (*Himantopus* and *Recurvirostra*) at the Ningxia site, and *Borrelia* in three bird species of Hebei (*Chlidonias leucopterus*, *Anser indicus*, and *Larus relictus*). Enteropathogenic *E. coli* (EPEC) was exclusively detected in *Chroicocephalus ridibundus* while typical EPEC (tEPEC, possessing the *eae* and *bfpA* genes) was found in Shandong only and atypical (aEPEC, possessing the *eae* gene but lacking the *bfpA* gene) in both Shandong and Yunnan.

**Table 2:** The detection rate (%) of 14 pathogens identified in migratory birds at ten habitat sites in China

	*Aeromonas*	*Plasmodium*	*Cryptosporidium*	*Entamoeba*	*Giardia*	EPEC	*C. jejuni/coli*	*Rickettsia*	*Borrelia*	HIV	*Plesiomonas*	*V.cholerae*	*T. gondii*	*E. faecalis*	Total
Fujian	-	-	2.3	-	-	-	-	-	-	-	-	-	-	-	2.3
Hebei	-	-	-	-	-	-	-	-	7.5	-	-	-	-	-	7.5
Heilongjiang	-	10.7	14.3	-	-	-	-	-	-	-	-	-	-	-	17.9
Hubei	51.6	16.1	9.7	35.5	19.4	-	3.2	-	-	-	-	-	-	-	87.1
Inner Mongolia	3.3	-	-	-	20.0	-	-	-	-	-	-	-	3.3	-	23.3
Ningxia	42.9	-	3.6	-	-	-	-	21.4	-	-	3.6	7.1	-	-	64.3
Qinghai	-	8.3	11.1	-	-	-	-	-	-	5.6	-	-	-	-	25.0
Shandong	10.7	-	7.1	10.7	3.6	25.0	3.6	-	-	-	-	-	-	-	35.7
Xizang	-	42.9	8.6	-	-	-	-	-	-	-	-	-	-	2.9	45.7
Yunnan	-	-	-	-	-	3.9	9.6	-	-	1.9	1.9	-	-	-	15.4
Total	9.1	7.4	5.1	4.0	3.7	2.6	2.0	1.7	0.9	0.9	0.6	0.6	0.3	0.3	30.5

EPEC included both typical EPEC and atypical EPEC

The pathogens in mixed infections were counted for each individual target

**Fig. 1:**
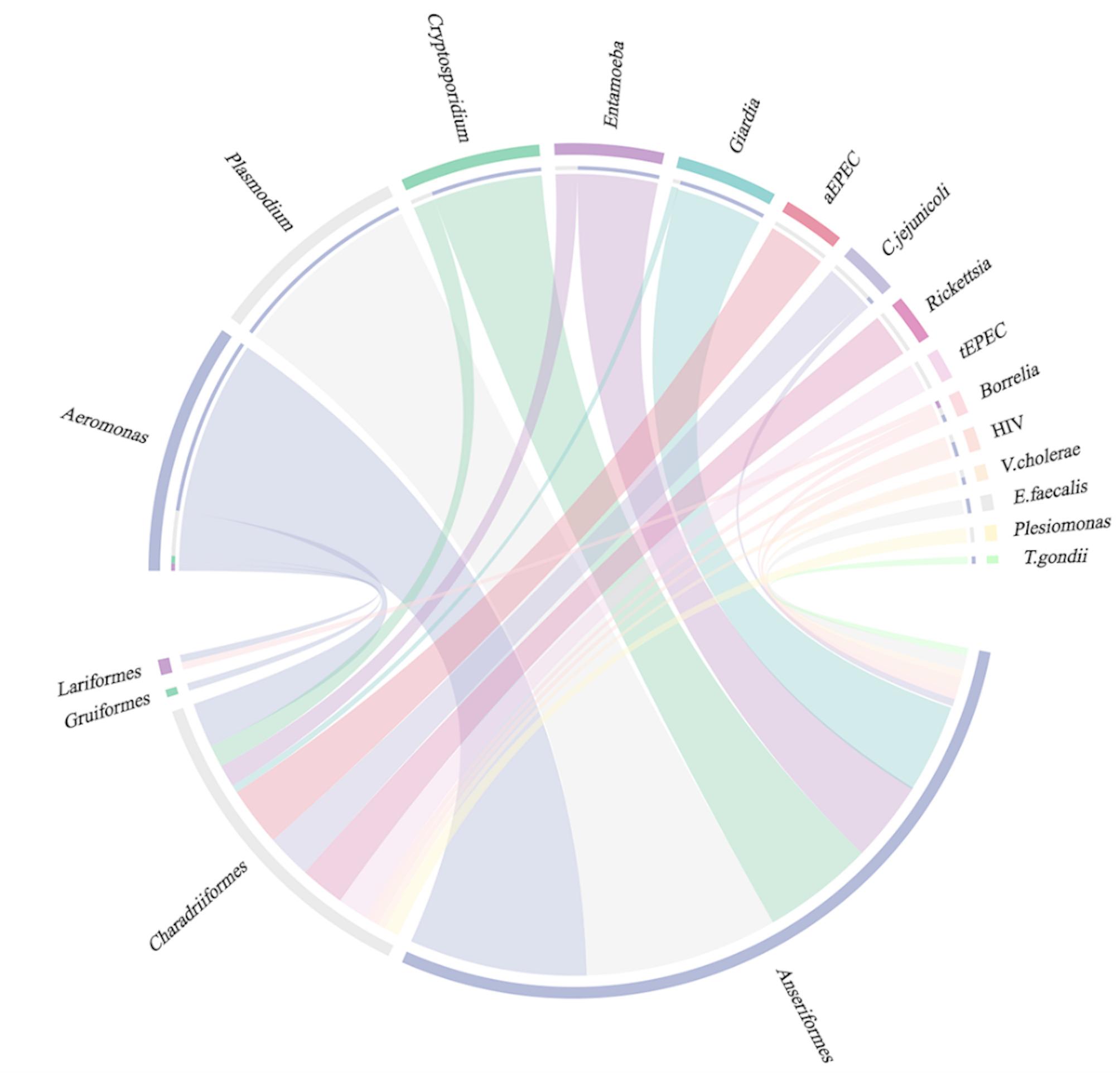
Distribution of the detected pathogens in the different migratory bird orders

Since majority of the samples were collected from *Anseriformes* and *Charadriiformes*, the pathogen distribution was further compared between these two orders (supplemental Figure S2). Overall, the pathogen detection rate was significantly higher in *Anseriformes* (39.1%, 72/184) than *Charadriiformes* (23.2%, 33/142, *p* < 0.05). The parasitic pathogens were more prevalent in *Anseriformes*, particularly with *Plasmodium* being exclusively detected in *Anseriformes* while the bacterial pathogens more prevalent in *Charadriiformes* (Fig. 1). Most of the water-borne pathogens, such as *Cryptosporidium*, *Giardia lamblia*, and *Aeromonas* were detected in *Anseriformes* (supplemental Figure S2). The foodborne pathogens, *Campylobacter* and enteropathogenic *E. coli*, including both tEPEC and aEPEC, were mostly detected in *Charadriiformes.* Mixed pathogens were detected in 25.0% (18/72) of the positive samples from *Anseriformes* and 21.2% (7/33) in *Charadriiformes* (supplemental Figure S2).

### Distribution of pathogens detected in *Anser* by season

**Fig. 2:**
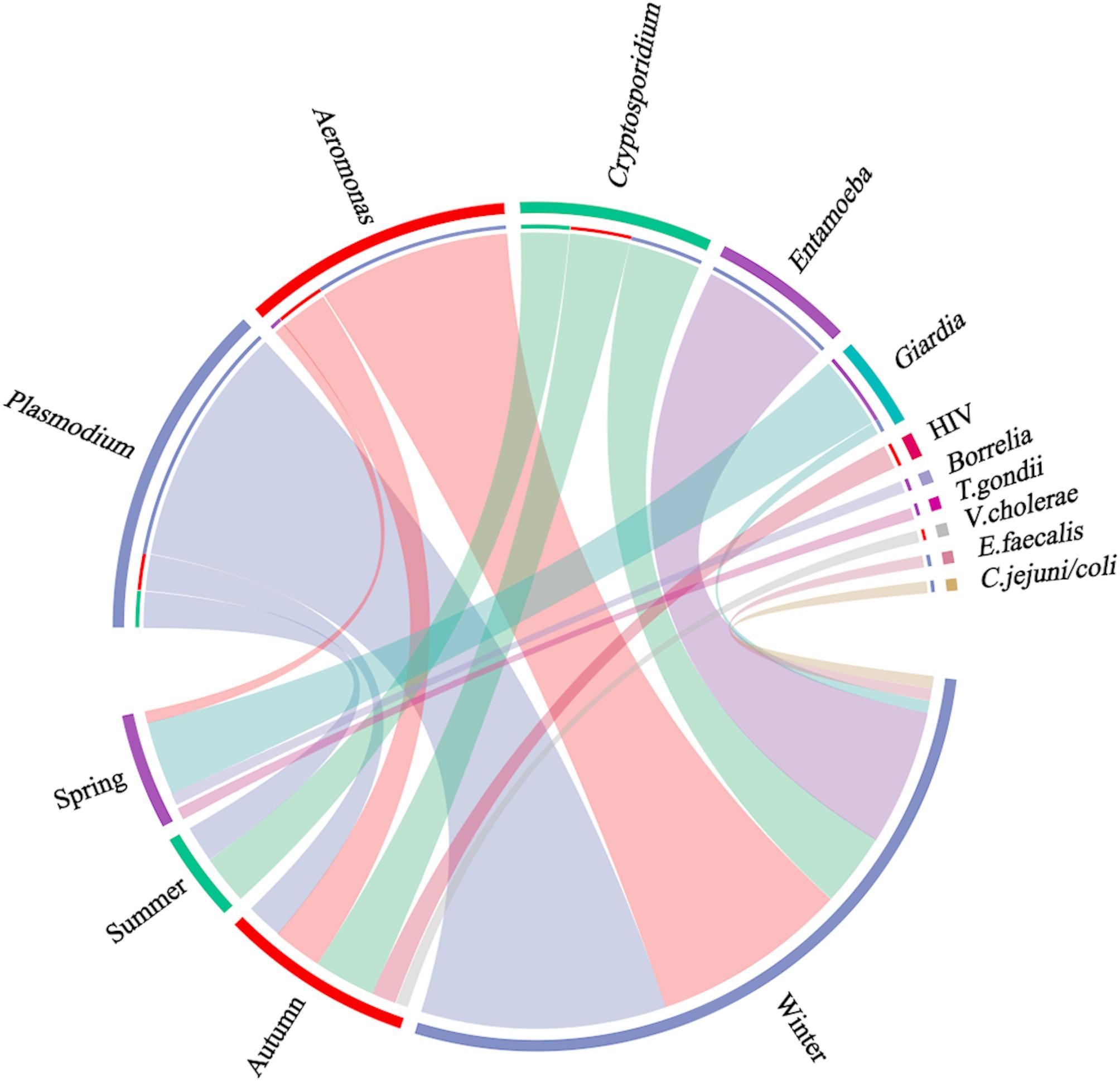
Distribution of pathogens detected in *Anser* by season

Among the 182 samples from *Anser*, most were collected from autumn/winter (68.1%, 124/182). The pathogen detection rate varied from 29.6% (8/27) in spring, 22.6% (7/31) for summer, 24.1% (14/58) for autumn, and 65.2% (43/66) for winter (*p* < 0.05). *Plasmodium*, *Aeromonas*, and *Entamoeba* were mostly detected in winter while *Cryptosporidium* distributed across summer, autumn, and winter. *Giardia lamblia* was mostly detected in spring (Fig. 2).

### Estimation of pathogen quantities

**Fig. 3:**
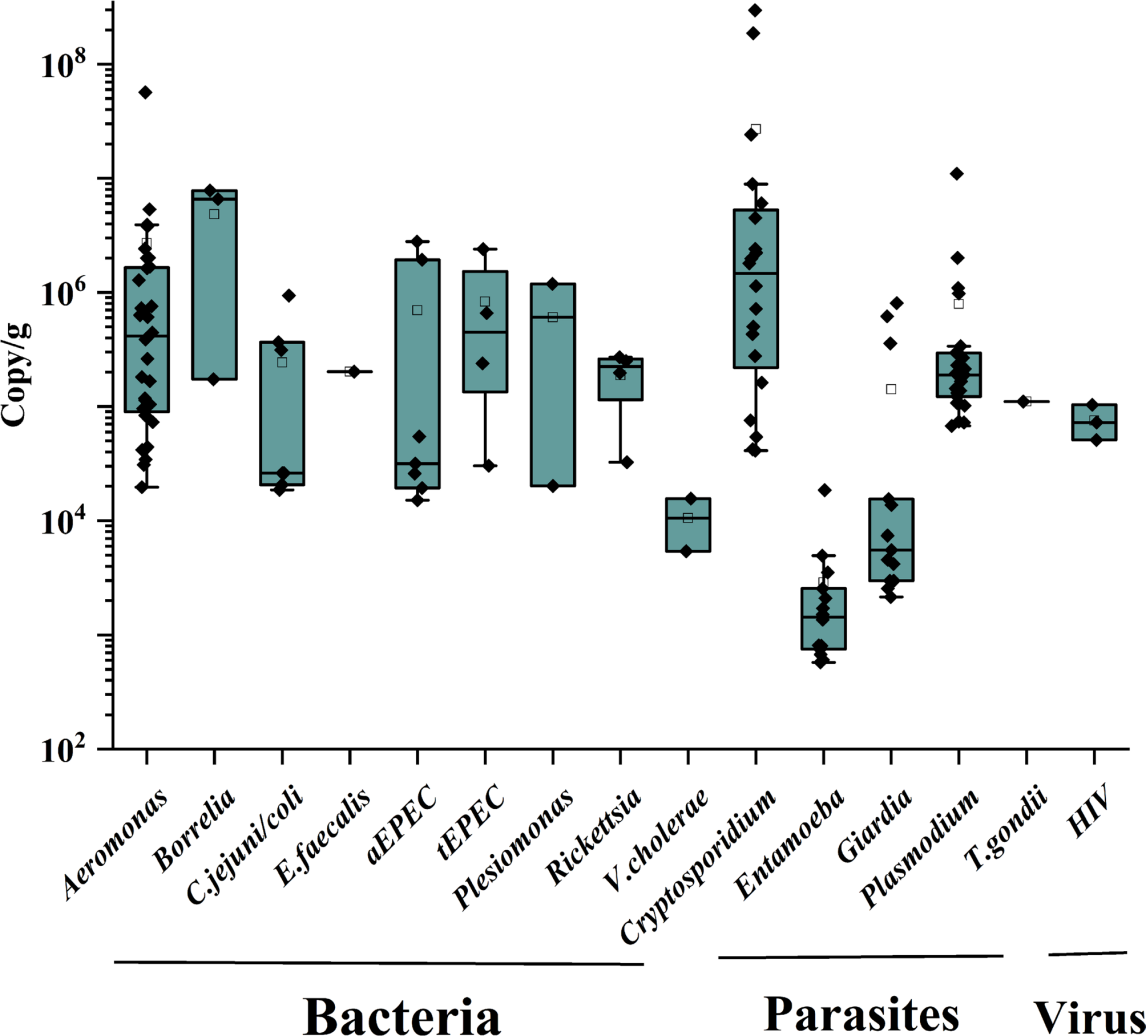
Pathogen quantities in the fecal samples of migratory birds determined by qPCR. The quantity was expressed as the copy number of gene per gram of feces

The pathogen quantity was calculated based on the Cq values using the standard curves. The results showed that the quantities ranged from 10^3^ to 2 × 10^8^ gene copies per gram of feces (Fig. 3). The bacterial pathogens were mostly present at 10^4^ to 10^7^ gene copies per gram of feces. The pathogenic loads of *Aeromonas* and *Plasmodium* in Hubei were higher than those in Ningxia and Xizang (*p* < 0.005), respectively.

### Detection and quantification of AMR genes

**Fig. 4:**
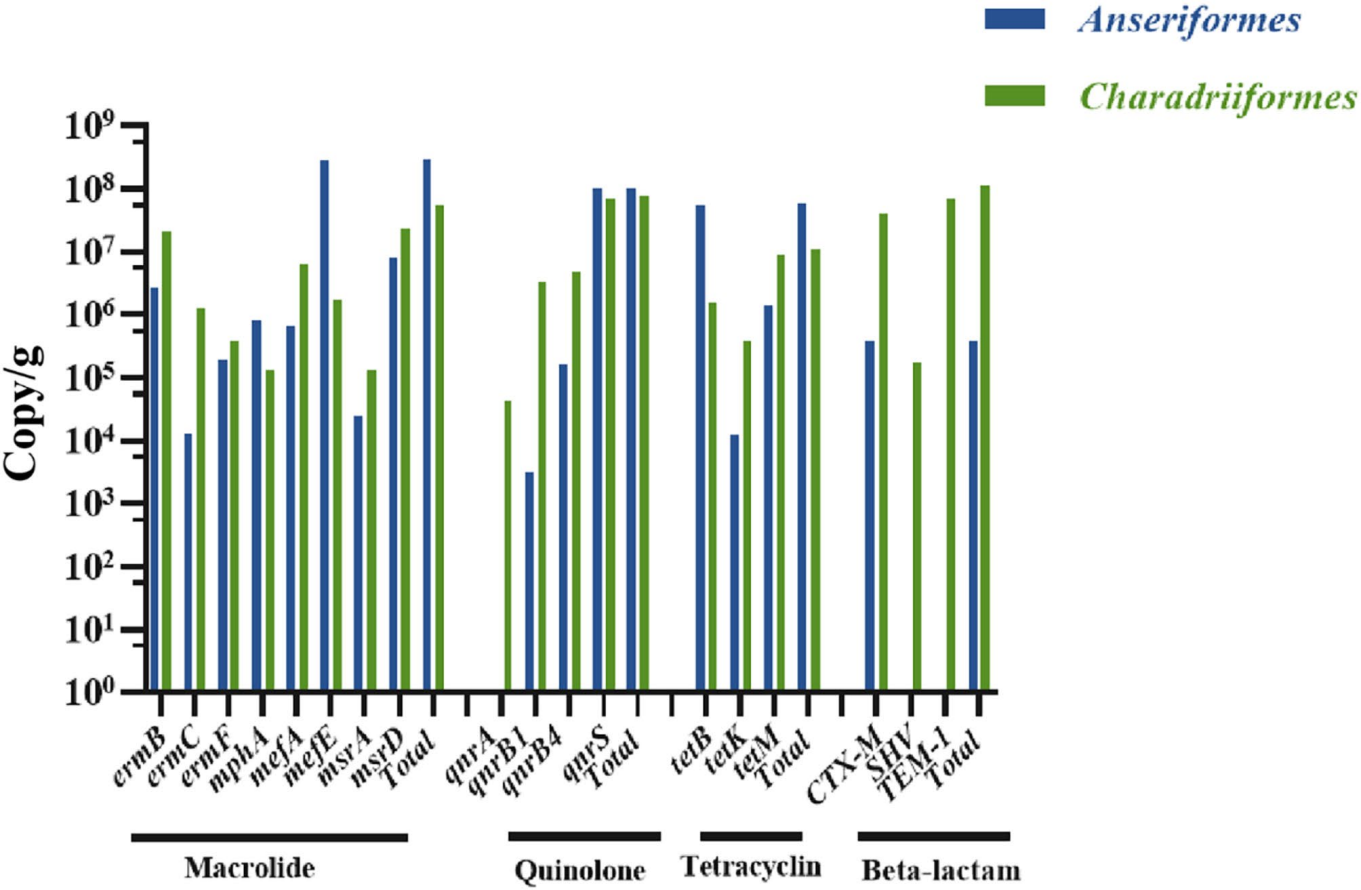
Averaged quantities of AMR genes detected in *Anseriformes* and *Charadriifornes*. The quantity of each gene was averaged among all the samples tested regardless of positivity for the relevant gene. The total quantity indicates the sum of the averaged quantities of the genes belonging to the same drug class

Of the 20 AMR genes associated with resistance to four classes of drugs including macrolides, quinolones, tetracyclines, and β-lactams, all were detected in 36 pooled feces from the migratory birds but two genes, i.e. *blaZ* and *ermA* (supplemental Table S2). Majority (30/36) of the pools were from *Anseriformes*, while six from *Charadriiformes*. Most of the pooled samples were positive for at least one AMR gene interrogated except 7 samples from *Anseriformes.* The quantities of each gene were calculated based on the Cq values using the standard curves, then averaged among the total number of samples tested, including the negatives, to estimate the overall exposure doses and compare between *Anseriformes* and *Charadriiformes.* Most of the genes were detected in both orders except *qnrA*, SHV, and TEM in only *Charadriiformes.* The averaged quantities were highly variable across genes even within the drug classes, from 10^3^ to > 10^8^ gene copies/gram of feces (Fig. 4). When summing copies of genes belonging to the same class, the total quantities were estimated to be 10^5^-10^9^ copies/gram of feces.

## Discussion

A multi-pathogen detection tool, TaqMan Array Card, was used in the current study to screen 99 pathogens and 20 AMR genes in the feces collected from a variety of migratory birds at different habitats of China. The interrogated pathogens were all related to human diseases and the qPCR assays used were previously developed, validated, and utilized for etiological and epidemiological analyses. The results showed that 30.5% of the samples were positive for at least one of the 14 pathogens identified. The distribution of these pathogens revealed region and bird type specificity, and certain seasonality. The most prevalent pathogens included water-borne *Aeromonas*, *Cryptosporidium*, and *Giardia lamblia*, food-borne *Campylobacter* and EPEC, and vector-borne *Plasmodium* and *Rickettsia*. This method greatly improved the detection efficiency and further increased the diversity of intestinal microorganisms and pathogens carried by migratory birds.

Per the recommendation from the World Health Organization (WHO), quantitative microbial risk assessment (QMRA) includes four steps, i.e. hazard identification, exposure assessment, dose-response modeling, and risk characterization [36]. qPCR based TAC platform enabled both detection and quantification of the pathogens. In the current study, the pathogen loads of bacteria ranged mostly between 20 and 1000 copies per mg of bird dropping. It is known that infection doses for some of the pathogens were low, such as 500–800 bacteria for *Campylobacter* [37]. Extensive estimation of the exposure doses of different pathogens in various hosts may be informative for more accurate risk assessment.

As expected, most of the pathogens were diarrhea associated enteropathogens. *Aeromonas* was the most common pathogen detected (9.1%, 32/351), predominantly in *Anseriformes*. About half of the migratory birds, mostly *Anser*, in Hubei and Ningxia carried *Aeromonas. Aeromonas* has previously been isolated from a variety of environmental sources, such as contaminated and drinking water, as well as tissues and body fluids of cold-blooded and warm-blooded animals [38–40]. It is commonly associated with sepsis in various aquatic organisms and gastrointestinal or parenteral diseases in humans [41]. The migration of waterfowl across national and intercontinental borders can provide a mechanism for the global spread of bacterial species. Halpern et al. identified three *Aeromonas* species in 19 birds of three different species with different abundance, suggesting that waterfowl had the potential to transmit *Aeromonas* across continents, thereby establishing new endemic areas far from the source of infection [42]. Liang et al. analyzed the drug resistance, virulence, and genetic diversity of *Aeromonas* in migratory birds from Guangxi, Guangdong, Ningxia, Jiangxi and Inner Mongolia in China, and the results showed that migratory birds carried high virulence and multidrug-resistant *Aeromonas* [43]. The current study revealed its geographical, seasonal, and host distribution, then the next step would be to delve deeper into its molecular characteristics to assess the potential threat to public health.

Notably, the most prevalent enteric parasite was *Cryptosporidium* (5.1%, 18/351), which is consistent with the findings from previously studies. *Cryptosporidium* has a broad host range, including humans, domestic animals, birds, fish, etc., and it is mainly transmitted by fecal-oral route [44]. As one of the leading causative agents of mortality due to diarrhea in children under 5, it is a ubiquitous water contaminant and widely transmitted [45–48]. A large-scale epidemiological survey of *Cryptosporidium* in chickens and ducks in Henan, China, by Wang et al. showed that infection rates varied from 3.4% in broilers to 16.3% in Pekin ducks [49], while the detection rate of *Cryptosporidium* was only 1.7% among migrating whooper swans in the same area [50]. The total prevalence rates of *Cryptosporidium* and *Giardia* reported by Jian et al. in wild birds in Qinghai Lake and the surrounding areas of the Tibetan Plateau in China were 8.98% and 3.39%, respectively [51]. The overall prevalence of *Cryptosporidium* in animal inhabiting drinking water catchments in three states across Australia was 18.3% [52]. Cautions have to be taken to select the proper PCR assays for *Cryptosporidium* detection due to high homology of ribosomal RNA gene, as the most often used target, between *Cryptosporidium* and algae, which is often present in environment samples and interfere the assay specificity. We further performed amplicon sequencing to validate the *Cryptosporidium* detection, confirmed its broadest distribution at seven of the ten sites, mostly from *Anser*.

Furthermore, *Cryptosporidium* and *Giardia* have been considered as important indicators for water quality due to the high stability and resistance of their oocysts in the environment [53–55]. In our study, *Cryptosporidium* or *Giardia lamblia* or both were detected in migratory birds, mostly from *Anseriformes*, inhabiting at lakes, swamps, and other wetlands, so their droppings likely affect the water quality. When humans, poultry, or other animals come into contact with these waters, it increases the risk and spread of relevant waterborne infectious diseases [56]. Therefore, monitoring pathogens, including other waterborne such as *Aeromonas*,* V. cholerae*, carried by migratory birds is essential for early warning of water pollution and it is also necessary to strengthen the eco-epidemiological research on the transmission dynamics in endemic areas and reduce the impact on human health.

The detection of other enteric pathogens showed geographical relevance. For example, *V. cholerae* was detected in Ningxia. *V. cholerae* O1 and O139 have been considered to be the only serotypes that cause diarrhea. However, recent studies demonstrated non-toxigenic non-O1/non-O139 *Vibrio cholerae* are also associated with diarrheal disease globally [57], indicating the importance of further characterization of these *V. cholerae* positives. *C. jejuni/coli* and EPEC were mostly detected in *Chroicocephalus ridibundus* in Shandong and Yunnan. Interestingly, aEPEC were present in birds from both regions while tEPEC only from Shandong, which likely reflected the difference in ecological environments.

In our study, some vector-borne pathogens were also detected in feces. *Plasmodium* was detected exclusively from *Anser* across four sites. Previous studies have suggested that migratory bird species might carry blood parasites with high diversity and prevalence. In addition, *Rickettsia* and *Borrelia*, as tick-borne pathogens, were detected in Ningxia and Hebei, respectively. Migratory birds, as hosts for ticks, may facilitate the spread of pathogens to new geographic areas through migration. Studies have shown that migratory birds may increase the risk of human exposure to ticks and their infections, especially during seasons of high tick activity [58–60]. Parker et al. in their analysis of tick and bird hosts showed that infection intensity was the greatest in birds captured during autumn migration [61]. In this study, six *Rickettsia*-positive samples were all collected from birds of *Anseriformes* in autumn in Ningxia while three *Borrelia* samples from different birds in Hebei. Hebei may serve as an important stopover site for birds along multiple migration routes, where a large number of birds congregate, potentially facilitating host exchange among birds. The reasons or mechanisms that such bloodstream pathogens were present in feces require further investigation.

As a demonstration of proof-of-concept, 20 AMR genes related to four important drug classes were tested along with the pathogens. Most were detected positive with the exposure dose mostly between 10^7^ and 10^8^ copies/gram of feces. Metagenomic sequencing has revealed a broad range of AMR genes in a variety of migratory birds [10]. By comparison (supplemental Table S2), our qPCR results revealed the presence of several AMR genes that were not identified by metagenomic sequencing, including *mphA*, *msrA*, and *msrD* for macrolide resistances, *qnrA*, *qnrB1*, *qnrB4*, and *qnrS* for quinolone resistances, CTX-M for β-lactam resistance. These discrepancies are worth further clarification because of the importance of these resistances in human health and poultry industry. Admittedly, only a small subset of the AMR genes was interrogated in the current study. Extensive and systematic targeted screening with such high throughput approach may provide more comprehensive understanding of the drug resistances carried and spread by migratory birds.

There are a few limitations in this study. Due to the sampling accessibility, temporal variation at a certain region was not captured, and the sample sizes for some migratory bird types were small. The individual specimens from a few pooled samples didn’t amplify for the relevant targets, leading to a potential underestimate of the detection. Alternative methods may be required to confirm these results. The presence of the pathogens was determined only by qPCR, without the confirmation of the viability or pathogenicity. While most of the targets were tested at the species level, several were at genus level, such as *Plasmodium*,* Rickettsia*,* Aeromonas*, etc. (supplemental Table S1). Currently targeted and metagenomic sequencing is ongoing to further characterize the genetic features, virulence, diversity, and host specificity of the detected pathogens, which can be novel species or strains as described previously [62]. The coverage of AMR gens was limited in the current study. More broad screening is needed for a comprehensive profile of drug resistance that can be spread by the migratory birds. Furthermore, similar high throughput strategy is being implemented to screen the environmental samples and those from poultry farms to explore the link and the potential transmission between the wild and domestic birds.

## Conclusions

In conclusion, TaqMan Array Card enabled high throughput screening of a broad range of pathogens and AMR genes in migratory birds to evaluate the potential hazardous effect to the environment. The advantage of this platform is modular, with the flexibility to accommodate additional targets including virulence genes, drug resistance genes, and any emerging agents, e.g. arboviruses such as Usutu virus. Further combined with a one health approach, future studies using such a tool would be of great value to estimate the spread risks from wildlife to domestic animals and humans. Rapid and long-term surveillance may provide guidance to implement targeted interventions to reduce the risk of pathogen transmission and zoonotic outbreaks.

## Electronic supplementary material

Below is the link to the electronic supplementary material.

Supplementary Material 1

## Data Availability

The sequences generated during the current study are deposited and available at NCBI, with the accession numbers PQ047601, PQ047618-PQ047627, PV124875-PV124878, PV124901-PV124936.

## Abbreviations

TAC: TaqMan Array Card
qPCR: Quantitative polymerase chain reaction
AMR: Antimicrobial resistance
EPEC: Enteropathogenic *Escherichia coli;*
tEPEC: Typical EPEC
aEPEC: Atypical EPEC
COI: Cytochrome oxidase sub gene I

## References

[1] Viana DS, Santamaría L, Figuerola J. Migratory birds as global dispersal vectors. Trends Ecol Evol. 2016;31:763–75. 10.1016/j.tree.2016.07.005. 27507683 10.1016/j.tree.2016.07.005

[2] Liu J, Xiao H, Lei F, et al. Highly pathogenic H5N1 influenza virus infection in migratory birds. Science. 2005;309:1206. 10.1126/science.1115273. 16000410 10.1126/science.1115273

[3] Pantin-Jackwood MJ, Costa-Hurtado M, Bertran K, et al. Infectivity, transmission and pathogenicity of H5 highly pathogenic avian influenza clade 2.3.4.4 (H5N8 and H5N2) united States index viruses in Pekin ducks and Chinese geese. Vet Res. 2017;48:33. 10.1186/s13567-017-0435-4. 28592320 10.1186/s13567-017-0435-4 PMC5463389

[4] Chan JF-W, To KK-W, Chen H, Yuen K-Y. Cross-species transmission and emergence of novel viruses from birds. Curr Opin Virol. 2015;10:63–9. 10.1016/j.coviro.2015.01.006 25644327 10.1016/j.coviro.2015.01.006 PMC7102742

[5] Jones KE, Patel NG, Levy MA, et al. Global trends in emerging infectious diseases. Nature. 2008;451:990–3. 10.1038/nature06536. 18288193 10.1038/nature06536 PMC5960580

[6] Dreyer S, Globig A, Bachmann L, et al. Longitudinal study on Extended-Spectrum Beta-Lactamase-E. Coli in Sentinel Mallard ducks in an important Baltic Stop-Over site for migratory ducks in Germany. Microorganisms. 2022;10:1968. 10.3390/microorganisms10101968. 36296245 10.3390/microorganisms10101968 PMC9612239

[7] Huang J, Zhou N, Cheng Z, et al. Chromosomally located BlaCMH in Enterobacter cloacae complex across human-bird-environment interfaces: A one-health perspective. Sci Total Environ. 2024;954:176486. 10.1016/j.scitotenv.2024.176486. 39322071 10.1016/j.scitotenv.2024.176486

[8] Olaru ID, Walther B, Schaumburg F. Zoonotic sources and the spread of antimicrobial resistance from the perspective of low and middle-income countries. Infect Dis Poverty. 2023;12:59. 10.1186/s40249-023-01113-z. 37316938 10.1186/s40249-023-01113-z PMC10265791

[9] Hwengwere K, Nair HP, Hughes KA, et al. Antimicrobial resistance in Antarctica: is it still a pristine environment? Microbiome. 2022;10:71. 10.1186/s40168-022-01250-x 35524279 10.1186/s40168-022-01250-x PMC9072757

[10] Cao J, Hu Y, Liu F, et al. Metagenomic analysis reveals the Microbiome and resistome in migratory birds. Microbiome. 2020;8:26. 10.1186/s40168-019-0781-8. 32122398 10.1186/s40168-019-0781-8 PMC7053137

[11] van Helden PD, van Helden LS, Hoal EG. One world, one health. EMBO Rep. 2013;14:497–501. 10.1038/embor.2013.61. 23681441 10.1038/embor.2013.61 PMC3674448

[12] Wang Q, Zhou Z-J, You Z, et al. Epidemiology and evolution of novel deltacoronaviruses in birds in central China. Transbound Emerg Dis. 2022;69:632–44. 10.1111/tbed.14029. 33559368 10.1111/tbed.14029 PMC8014545

[13] Zhang X, Li Y, Jin S, et al. H9N2 influenza virus spillover into wild birds from poultry in China bind to human-type receptors and transmit in mammals via respiratory droplets. Transbound Emerg Dis. 2022;69:669–84. 10.1111/tbed.14033. 33566453 10.1111/tbed.14033

[14] Zhang Z, Zhou H, Cao H, et al. Human-to-human transmission of Chlamydia psittaci in China, 2020: an epidemiological and aetiological investigation. Lancet Microbe. 2022;3:e512–20. 10.1016/S2666-5247(22)00064-7. 35617977 10.1016/S2666-5247(22)00064-7

[15] Chu Y, Wang D, Hao W, et al. Prevalence, antibiotic resistance, virulence genes and molecular characteristics of Salmonella isolated from ducks and wild geese in China. Food Microbiol. 2024;118:104423. 10.1016/j.fm.2023.104423. 38049277 10.1016/j.fm.2023.104423

[16] Liao F, Gu W, Li D, et al. Characteristics of microbial communities and intestinal pathogenic bacteria for migrated Larus ridibundus in Southwest China. Microbiologyopen. 2019;8:e00693. 10.1002/mbo3.693. 29978594 10.1002/mbo3.693 PMC6460275

[17] Madut DB, Rubach MP, Allan KJ, et al. Epidemiologic and genomic characterization of an outbreak of rift Valley fever among humans and dairy cattle in Northern Tanzania. J Infect Dis Jiae. 2024;562. 10.1093/infdis/jiae562. 10.1093/infdis/jiae562 PMC12069657 39535803

[18] Kang M, Wang L-F, Sun B-W, et al. Zoonotic infections by avian influenza virus: changing global epidemiology, investigation, and control. Lancet Infect Dis. 2024;24:e522–31. 10.1016/S1473-3099(24)00234-2. 38878787 10.1016/S1473-3099(24)00234-2

[19] Reed KD, Meece JK, Henkel JS, Shukla SK. Birds, migration and emerging zoonoses: West nile virus, Lyme disease, influenza A and enteropathogens. Clin Med Res. 2003;1:5–12. 10.3121/cmr.1.1.5. 15931279 10.3121/cmr.1.1.5 PMC1069015

[20] Sonawane GG, Tripathi BN. Comparison of a quantitative real-time polymerase chain reaction (qPCR) with conventional PCR, bacterial culture and ELISA for detection of Mycobacterium avium subsp. Paratuberculosis infection in sheep showing pathology of Johne’s disease. Springerplus. 2013;2:45. 10.1186/2193-1801-2-45. 23539663 10.1186/2193-1801-2-45 PMC3604594

[21] Lappan R, Jirapanjawat T, Williamson DA, et al. Simultaneous detection of multiple pathogens with the TaqMan array card. MethodsX. 2022;9:101707. 10.1016/j.mex.2022.101707. 35518918 10.1016/j.mex.2022.101707 PMC9062751

[22] Liu J, Gratz J, Amour C, et al. A laboratory-developed TaqMan array card for simultaneous detection of 19 enteropathogens. J Clin Microbiol. 2013;51:472–80. 10.1128/JCM.02658-12. 23175269 10.1128/JCM.02658-12 PMC3553916

[23] Moore CC, Jacob ST, Banura P, et al. Etiology of Sepsis in Uganda using a quantitative polymerase chain Reaction-based TaqMan array card. Clin Infect Dis. 2019;68:266–72. 10.1093/cid/ciy472. 29868873 10.1093/cid/ciy472 PMC6321855

[24] Kodani M, Yang G, Conklin LM, et al. Application of TaqMan low-density arrays for simultaneous detection of multiple respiratory pathogens. J Clin Microbiol. 2011;49:2175–82. 10.1128/JCM.02270-10. 21471348 10.1128/JCM.02270-10 PMC3122721

[25] Harvey JJ, Chester S, Burke SA, et al. Comparative analytical evaluation of the respiratory TaqMan array card with real-time PCR and commercial multi-pathogen assays. J Virol Methods. 2016;228:151–7. 10.1016/j.jviromet.2015.11.020. 26640122 10.1016/j.jviromet.2015.11.020 PMC7113746

[26] Vieira CB, Araújo IT, Ferreira FC, et al. Fast screening of enteropathogens in marine water samples. Braz J Microbiol. 2022;53:1439–46. 10.1007/s42770-022-00770-w. 35596892 10.1007/s42770-022-00770-w PMC9433501

[27] Liu J, Ochieng C, Wiersma S, et al. Development of a TaqMan array card for Acute-Febrile-Illness outbreak investigation and surveillance of emerging pathogens, including Ebola virus. J Clin Microbiol. 2016;54:49–58. 10.1128/JCM.02257-15. 26491176 10.1128/JCM.02257-15 PMC4702733

[28] Liu J, Platts-Mills JA, Juma J, et al. Use of quantitative molecular diagnostic methods to identify causes of diarrhoea in children: a reanalysis of the GEMS case-control study. Lancet. 2016;388:1291–301. 10.1016/S0140-6736(16)31529-X. 27673470 10.1016/S0140-6736(16)31529-X PMC5471845

[29] Marks F, Liu J, Soura AB, et al. Pathogens that cause acute febrile illness among children and adolescents in Burkina Faso, Madagascar, and Sudan. Clin Infect Dis. 2021;73:1338–45. 10.1093/cid/ciab289. 33822011 10.1093/cid/ciab289 PMC8528393

[30] Brennhofer SA, Rogawski McQuade ET, Zhang J, et al. Effect of biannual Azithromycin to children under 5 years on the carriage of respiratory pathogens among children aged 7–11 years. Am J Trop Med Hyg. 2023;108:428–32. 10.4269/ajtmh.22-0583. 36535258 10.4269/ajtmh.22-0583 PMC9896336

[31] Pholwat S, Liu J, Taniuchi M, et al. Genotypic antimicrobial resistance assays for use on E. coli isolates and stool specimens. PLoS ONE. 2019;14:e0216747. 10.1371/journal.pone.0216747. 31075137 10.1371/journal.pone.0216747 PMC6510447

[32] Spano F, Putignani L, McLauchlin J, et al. PCR-RFLP analysis of the Cryptosporidium oocyst wall protein (COWP) gene discriminates between C. wrairi and C. parvum, and between C. parvum isolates of human and animal origin. FEMS Microbiol Lett. 1997;150. 10.1016/s0378-1097(97)00115-8. 10.1016/s0378-1097(97)00115-8 9170264

[33] Sulaiman IM, Lal AA, Xiao L. Molecular phylogeny and evolutionary relationships of Cryptosporidium parasites at the actin locus. J Parasitol. 2002;88:388–94. 10.1645/0022-3395(2002)088[0388:MPAERO]2.0.CO;2. 12054017 10.1645/0022-3395(2002)088[0388:MPAERO]2.0.CO;2

[34] de Melo AA, Nunes R, Telles MPD. Same information, new applications: revisiting primers for the avian COI gene and improving DNA barcoding identification. Org Divers Evol. 2021;21:599–614. 10.1007/s13127-021-00507-x

[35] Staggs SE, Beckman EM, Keely, SP, et al. The applicability of TaqMan-Based quantitative Real-Time PCR assays for detecting and enumerating Cryptosporidium spp. Oocysts in the environment. PLoS ONE. 201 10.1371/journal.pone.0066562 10.1371/journal.pone.0066562 PMC3689768 23805235

[36] Cao SK, Jiang YY, Yuan ZY, et al. Quantitative microbial risk assessment of Cryptosporidium and giardia in public drinking water in China. Biomed Environ Sci. 2021;34:493–8. 10.3967/bes2021.068. 34284858 10.3967/bes2021.068

[37] Janssen R, Krogfelt KA, Cawthraw SA, et al. Host-pathogen interactions in Campylobacter infections: the host perspective. Clin Microbiol Rev. 2008;21:505–18. 10.1128/CMR.00055-07. 18625685 10.1128/CMR.00055-07 PMC2493085

[38] Abd El-Ghany WA. A review on aeromoniasis in poultry: A bacterial disease of zoonotic nature. J Infect Dev Ctries. 2023;17:1–9. 10.3855/jidc.17186. 36795920 10.3855/jidc.17186

[39] Martínez-Murcia AJ, Saavedra MJ, Mota VR, et al. Aeromonas aquariorum Sp. nov., isolated from Aquaria of ornamental fish. Int J Syst Evol Microbiol. 2008;58:1169–75. 10.1099/ijs.0.65352-0. 18450708 10.1099/ijs.0.65352-0

[40] Govender R, Amoah ID, Adegoke A A, et al. Identification, antibiotic resistance, and virulence profiling of Aeromonas and Pseudomonas species from wastewater and surface water. Environ Monit Assess 10.1007/s10661-021-09046-6 10.1007/s10661-021-09046-6 33893564

[41] Bhowmick UD, Bhattacharjee S. Bacteriological, clinical and virulence aspects of Aeromonas-associated diseases in humans. Pol J Microbiol. 2018;67:137–49. 10.21307/pjm-2018-020. 30015452 10.21307/pjm-2018-020 PMC7256846

[42] Halpern M, Senderovich Y, Izhaki I. Waterfowl: the missing link in epidemic and pandemic cholera dissemination? PLoS Pathog. 2008;4:e1000173. 10.1371/journal.ppat.1000173. 18974827 10.1371/journal.ppat.1000173 PMC2565833

[43] Liang B, Ji X, Jiang B, et al. Virulence, antibiotic resistance, and phylogenetic relationships of Aeromonas spp. Carried by migratory birds in China. Microorganisms. 2022;11:7. 10.3390/microorganisms11010007. 36677299 10.3390/microorganisms11010007 PMC9862355

[44] Ryan U. Cryptosporidium in birds, fish and amphibians. Exp Parasitol. 2010;124:113–20. 10.1016/j.exppara.2009.02.002. 19545515 10.1016/j.exppara.2009.02.002

[45] Kotloff KL, Nataro JP, Blackwelder WC, et al. Burden and aetiology of diarrhoeal disease in infants and young children in developing countries (the global enteric multicenter study, GEMS): a prospective, case-control study. Lancet. 2013;382:209–22. 10.1016/S0140-6736(13)60844-2. 23680352 10.1016/S0140-6736(13)60844-2

[46] GBD Diarrhoeal Diseases Collaborators. Estimates of global, regional, and National morbidity, mortality, and aetiologies of diarrhoeal diseases: a systematic analysis for the global burden of disease study 2015. Lancet Infect Dis. 2017;17:909–48. 10.1016/S1473-3099(17)30276-1. 28579426 10.1016/S1473-3099(17)30276-1 PMC5589208

[47] Fayer R. Cryptosporidium: a water-borne zoonotic parasite. Vet Parasitol. 2004;126:37–56. 10.1016/j.vetpar.2004.09.004. 15567578 10.1016/j.vetpar.2004.09.004

[48] Liu A, Gong B, Liu X, et al. A retrospective epidemiological analysis of human Cryptosporidium infection in China during the past three decades (1987–2018). PLoS Negl Trop Dis. 2020;14:e0008146. 10.1371/journal.pntd.0008146. 32226011 10.1371/journal.pntd.0008146 PMC7145189

[49] Wang R, Jian F, Sun Y, et al. Large-scale survey of Cryptosporidium spp. In chickens and Pekin ducks (Anas platyrhynchos) In Henan, China: prevalence and molecular characterization. Avian Pathol. 2010;39:447–51. 10.1080/03079457.2010.518314. 21154053 10.1080/03079457.2010.518314

[50] Wang K, Gazizova A, Wang Y, et al. First detection of Cryptosporidium spp. In migratory whooper swans (Cygnus cygnus) In China. Microorganisms. 2019;8. 10.3390/microorganisms8010006 10.3390/microorganisms8010006 PMC7023085 31861389

[51] Jian Y, Zhang X, Li X, et al. Occurrence of Cryptosporidium and giardia in wild birds from Qinghai lake on the Qinghai-Tibetan plateau, China. Parasitol Res. 2021;120:615–28. 10.1007/s00436-020-06993-w. 33415392 10.1007/s00436-020-06993-w

[52] Zahedi A, Monis P, Gofton AW, et al. Cryptosporidium species and subtypes in animals inhabiting drinking water catchments in three States across Australia. Water Res. 2018;134:327–40. 10.1016/j.watres.2018.02.005 29438893 10.1016/j.watres.2018.02.005

[53] Bouzid M, Hunter PR, Chalmers RM, Tyler KM. Cryptosporidium pathogenicity and virulence. Clin Microbiol Rev. 2013;26:115–34. 10.1128/CMR.00076-12. 23297262 10.1128/CMR.00076-12 PMC3553671

[54] Monis PT, Thompson RCA. Cryptosporidium and Giardia-zoonoses: fact or fiction? Infection. Genet Evol. 2003;3:233–44. 10.1016/j.meegid.2003.08.003. 10.1016/j.meegid.2003.08.003 14636685

[55] Kassa H, Harrington BJ, Bisesi MS. Cryptosporidiosis: a brief literature review and update regarding Cryptosporidium in feces of Canada geese (Branta canadensis). J Environ Health. 2004;66:34–40. 15032111

[56] Solarczyk P, Wojtkowiak-Giera A, Heddergott M. Migrating Anatidae as sources of environmental contamination with zoonotic Giardia, Cryptosporidium, cyclospora and microsporidia. Pathogens. 2023;12:487. 10.3390/pathogens12030487. 36986409 10.3390/pathogens12030487 PMC10057910

[57] Jacqueline C, Soto SR, Herrera-Leon S. (2024) Non-toxigenic cases of Vibrio cholerae in Spain from 2012 to 2022. Microbial genomics 10:. 10.1099/mgen.0.001315 10.1099/mgen.0.001315 PMC11633944 39661068

[58] Hasle G. Transport of Ixodid ticks and tick-borne pathogens by migratory birds. Front Cell Infect Microbiol. 2013;3:48. 10.3389/fcimb.2013.00048. 24058903 10.3389/fcimb.2013.00048 PMC3767891

[59] Michelet L, Joncour G, Devillers E, et al. Tick species, tick-borne pathogens and symbionts in an insular environment off the Coast of Western France. Ticks tick-borne Dis. 2016;7. 10.1016/j.ttbdis.2016.08.014 10.1016/j.ttbdis.2016.08.014 27622976

[60] Lommano E, Dvořák C, Vallotton L, et al. Tick-borne pathogens in ticks collected from breeding and migratory birds in Switzerland. Ticks Tick Borne Dis. 2014;5:871–82. 10.1016/j.ttbdis.2014.07.001. 25113989 10.1016/j.ttbdis.2014.07.001

[61] Parker CM, Miller JR, Allan BF. Avian and habitat characteristics influence tick infestation among birds in Illinois. J Med Entomol. 2017;54:550–8. 10.1093/jme/tjw235. 28399205 10.1093/jme/tjw235

[62] Wu S, Jia R, Wang Y, et al. Prevalence, diversity, and virulence of Campylobacter carried by migratory birds at four major habitats in China. Pathogens (Basel Switzerland). 2024;13. 10.3390/pathogens13030230 10.3390/pathogens13030230 PMC10975922 38535573

