# Supplementary material for: High throughput screening for human disease associated-pathogens and antimicrobial resistance genes in migratory birds at ten habitat sites in China
Source: BMC Microbiol. 2025 Jun 6;25:355. doi: 10.1186/s12866-025-04059-4 (PMC12143040; doi:10.1186/s12866-025-04059-4)
Supplement: Supplementary file 1 — Supplementary Material 1 [file 12866_2025_4059_MOESM1_ESM.docx]

Supplemental Table S1. Pathogens interrogated by the three customized TaqMan Array Cards used in this study. These pathogens are involved in enteric, respiratory, and bloodstream infections, respectively.

| Pathogens | Type | Enteric infection | Respiratory infection | Bloodstream infection | Reported in avian | Detected in this study | Reference for qPCR assay |
| --- | --- | --- | --- | --- | --- | --- | --- |
| Acinetobacter baumannii | Bacterium |  | √ | √ | [1, 2] |  | [3] |
| Aeromonas | Bacterium | √ |  |  | [4, 5] | √ | [6] |
| Bacillus anthracis | Bacterium |  |  | √ | [7] |  | [8] |
| Bacteroides fragilis | Bacterium | √ |  |  | [9] |  | [6] |
| Bartonella | Bacterium |  |  | √ | [10, 11] |  | [12] |
| Bordetella parapertussis | Bacterium |  | √ |  |  |  | [13] |
| Bordetella pertussis | Bacterium |  | √ |  |  |  | [14] |
| Borrelia | Bacterium |  |  | √ | [15] | √ | [16] |
| Brucella | Bacterium |  |  | √ | [17] |  | [12] |
| Burkholderia pseudomallei | Bacterium |  | √ |  | [18] |  | [19] |
| Campylobacter jejuni/coli | Bacterium | √ |  |  | [20, 21] | √ | [6] |
| Chlamydia pneumoniae | Bacterium |  | √ |  |  |  | [13] |
| Chlamydia psittaci | Bacterium |  |  | √ | [22] |  | [23] |
| Clostridium difficile | Bacterium | √ |  |  | [24] |  | [6] |
| Coxiella burnetii | Bacterium |  |  | √ | [25] |  | [12] |
| Enteroaggregative *E. coli* | Bacterium | √ |  |  |  |  | [6] |
| Enterococcus faecalis | Bacterium |  |  | √ | [26] | √ | [27] |
| Enteropathogenic *E. coli* | Bacterium | √ |  |  | [28] | √ | [6] |
| Enterotoxigenic *E. coli* | Bacterium | √ |  |  | [28] | √ | [6] |
| Escherichia albertii | Bacterium | √ |  |  | [29] |  | [30] |
| Haemophilus influenzae | Bacterium |  | √ | √ |  |  | [3] |
| Helicobacter pylori | Bacterium | √ |  |  |  |  | [6] |
| Klebsiella oxytoca | Bacterium |  |  | √ |  |  | [3] |
| Klebsiella pneumoniae | Bacterium |  | √ | √ | [31] |  | [3] |
| Legionella | Bacterium |  | √ |  |  |  | [32] |
| Leptospira interrogans | Bacterium |  |  | √ |  |  | [12] |
| Listeria monocytogenes | Bacterium |  |  | √ | [17] |  | [33] |
| Moraxella catarrhalis | Bacterium |  | √ |  |  |  | [14] |
| Mycobacterium avium complex | Bacterium |  | √ | √ | [34, 35] |  | [3] |
| Mycobacterium tuberculosis | Bacterium |  | √ | √ | [36] |  | [3] |
| Mycoplasma pneumoniae | Bacterium |  | √ |  |  |  | [14] |
| Neisseria meningitidis | Bacterium |  |  | √ |  |  | [3] |
| Orientia tsutsugamushi | Bacterium |  |  | √ |  |  | [3] |
| Plesiomonas shigelloides | Bacterium | √ |  |  | [37] | √ | [6] |
| Pseudomonas aeruginosa | Bacterium |  | √ |  | [38] |  | [3] |
| Rickettsia | Bacterium |  |  | √ | [39] | √ | [12] |
| Salmonella enterica | Bacterium | √ |  | √ | [17] |  | [6] |
| Shiga toxin producing *E. coli* | Bacterium | √ |  |  | [28] |  | [6] |
| Shigella/enteroinvasive *E. coli* | Bacterium | √ |  |  | [40] |  | [6] |
| Staphylococcus aureus | Bacterium |  | √ | √ | [31] |  | [3] |
| Streptococcus agalactiae | Bacterium |  |  | √ |  |  | [3] |
| Streptococcus pneumoniae | Bacterium |  | √ | √ |  |  | [3] |
| Streptococcus pyogenes | Bacterium |  | √ | √ |  |  | [3] |
| Ureaplasma | Bacterium |  | √ |  | [41] |  | [33] |
| Vibrio cholerae | Bacterium | √ |  |  | [42] | √ | [6] |
| Vibrio parahaemolyticus | Bacterium | √ |  |  | [43] |  | [6] |
| Yersinia enterocolitica | Bacterium | √ |  |  | [44] |  | [6] |
| Yersinia pestis | Bacterium |  |  | √ | [34] |  | [12] |
| Candida | Fungus |  |  | √ | [45] |  | [3] |
| Cryptococcus neoformans | Fungus |  |  | √ | [46] |  | [3] |
| Encephalitozoon intestinalis | Fungus | √ |  |  | [25] |  | [6] |
| Enterocytozoon bieneusi | Fungus | √ |  |  | [47] |  | [6] |
| Histoplasma capsulatum | Fungus |  |  | √ | [48] |  | [3] |
| Pneumocystis jirovecii | Fungus |  | √ |  |  |  | [14] |
| Ancylostoma duodenale | Parasite | √ |  |  | [49] |  | [6] |
| Ascaris lumbricoides | Parasite | √ |  |  |  |  | [6] |
| Blastocystis hominis | Parasite | √ |  |  | [50] |  | [6] |
| Cryptosporidium | Parasite | √ |  |  | [51] | √ | [6] |
| Cyclospora cayetanensis | Parasite | √ |  |  | [52] |  | [6] |
| Cystoisospora belli | Fungus | √ |  |  |  |  | [6] |
| Entamoeba histolytica and pan | Parasite | √ |  |  | [53] | √ | [6] |
| Giardia lamblia | Parasite | √ |  |  | [54] | √ | [6] |
| Hymenolepis nana | Parasite | √ |  |  | [55] |  | [56] |
| Leishmania | Parasite |  |  | √ |  |  | [12] |
| Necator americanus | Parasite | √ |  |  |  |  | [6] |
| Plasmodium | Parasite |  |  | √ | [57] | √ | [12] |
| Schistosoma | Parasite |  |  | √ | [58] |  | [6] |
| Strongyloides stercoralis | Parasite | √ |  |  |  |  | [6] |
| Toxoplasma gondii | Parasite |  |  | √ | [59] | √ | [3] |
| Trichuris trichiura | Parasite | √ |  |  |  |  | [6] |
| Trypanosoma brucei | Parasite |  |  | √ | [60] |  | [12] |
| Trypanosoma cruzi | Parasite |  |  | √ | [61] |  | [16] |
| Adenovirus 40/41 and Pan | Virus | √ | √ | √ | [62, 63] |  | [6] |
| Astrovirus | Virus | √ |  |  | [64] |  | [6] |
| Bocavirus | Virus |  | √ |  | [50] |  | [14] |
| Chikungunya | Virus |  |  | √ |  |  | [12] |
| Coronavirus (229E, HKU1, NL63, OC43, SARS, MERS, SARS-CoV-2) | Virus |  | √ |  | [65]  [66] |  | [67, 68] |
| Crimean-Congo hemorrhagic fever virus | Virus |  |  | √ | [69] |  | [12] |
| Cytomegalovirus | Virus |  | √ | √ |  |  | [6] |
| Dengue | Virus |  |  | √ | [70] |  | [12] |
| Enterovirus | Virus | √ |  | √ |  |  | [6] |
| Hepatitis E | Virus |  |  | √ | [71, 72] |  | [12] |
| Herpes simplex virus 1 and 2 | Virus |  |  | √ |  |  | [3] |
| human immunodeficiency virus I and II | Virus |  |  | √ | [48] | √ | [3] |
| Human metapneumovirus | Virus |  | √ |  | [73] |  | [14] |
| Influenza A and B | Virus |  | √ |  | [74] |  | [14] |
| Mayaro virus | Virus |  |  | √ |  |  | [16] |
| Nipah | Virus |  |  | √ |  |  | [12] |
| Norovirus GI and Gll | Virus | √ |  |  |  |  | [6] |
| O'nyong nyong virus | Virus |  | √ |  | [75] |  | [12] |
| Parainfluenza 1-4 | Virus |  | √ |  |  |  | [14] |
| Parechovirus | Virus |  | √ |  | [76] |  | [32] |
| Respiratory Syncytial Virus | Virus |  | √ |  |  |  | [14] |
| Rhinovirus | Virus |  | √ |  |  |  | [14] |
| Rotavirus | Virus | √ |  |  | [77] |  | [6] |
| Sapovirus | Virus | √ |  |  |  |  | [6] |
| West Nile virus | Virus |  |  | √ | [78] |  | [12] |
| Yellow Fever virus | Virus |  | √ |  | [79] |  | [12] |
| Zika virus | Virus |  |  | √ |  |  | [3] |

Supplemental Table S2 AMR genes included in the current study and their identification in metagenomic sequencing study[80].

| AMR genes | Drug class | Detection in metagenomic sequencing (No. detected/Total sample size)[80] | | | | | | | | | | | Detection in the current study | |
| --- | --- | --- | --- | --- | --- | --- | --- | --- | --- | --- | --- | --- | --- | --- |
|  |  | Yes/No | A. indicus | G. grus | A. fabalis | C. cygnus | T. tadorna | A. alba | T. nebularia | A. cygnoides | T. ferruginea | A. anser | Yes/No | In Anser |
| ermA  ermB  ermC  ermF  mphA  mefA  mefE  msrA  msrD | Macrolides | Yes  Yes  Yes  Yes  No  Yes  Yes  No  No | 1/12  8/12  1/12  3/12  -  4/12  0/12  -  - | 0/11  3/11  0/11  4/11  -  6/11  4/11  -  - | 0/10  0/10  0/10  0/10  -  0/10  1/10  -  - | 6/15  14/15  5/15  15/15  -  15/15  0/15  -  - | 0/10  1/10  0/10  3/10  -  2/10  0/10  -  - | 0/5  2/5  1/5  4/5  -  5/5  0/5  -  - | 8/8  8/8  7/8  8/8  -  8/8  0/8  -  - | 0/14  3/14  1/14  9/14  -  3/14  0/14  -  - | 0/2  0/2  0/2  0/2  -  1/2  0/2  -  - | 0/13  3/13  1/13  1/13  -  2/13  0/13  -  - | No  Yes  Yes  Yes  Yes  Yes  Yes  Yes  Yes | No  Yes  Yes  Yes  Yes  Yes  Yes  Yes  Yes |
| qnrA  qnrB1  qnrB4  qnrS | Quinolones | No  No  No  No | -  -  -  - | -  -  -  - | -  -  -  - | -  -  -  - | -  -  -  - | -  -  -  - | -  -  -  - | -  -  -  - | -  -  -  - | -  -  -  - | Yes  Yes  Yes  Yes | No  Yes  Yes  Yes |
| tetB  tetK  tetM | Tetracyclines | Yes  Yes  Yes | 2/12  5/12  7/12 | 6/11  2/11  9/11 | 1/10  0/10  2/10 | 15/15  14/15  15/15 | 5/10  1/10  1/10 | 3/5  1/5  0/5 | 8/8  8/8  8/8 | 7/14  2/14  1/14 | 0/2  0/2  0/2 | 1/13  1/13  0/13 | Yes  Yes  Yes | Yes  Yes  Yes |
| blaZ  CTX-M  SHV  TEM | β-lactams | No  No  Yes  Yes | -  -  0/12  9/12 | -  -  2/11  8/11 | -  -  0/10  3/10 | -  -  0/15  11/15 | -  -  0/10  5/10 | -  -  0/5  5/5 | -  -  0/8  7/8 | -  -  0/14  6/14 | -  -  0/2  2/2 | -  -  2/13  10/13 | No  Yes  Yes  Yes | No  Yes  No  No |

-, not applicable.

Supplemental Table S3 Target Genes and Primer Sequences for *Cryptosporidium* Detection.

| Target Gene | Sequence（5’-3’） | Amplicon Size |
| --- | --- | --- |
| 18S rRNA qPCR | F：GGGTTGTATTTATTAGATAAAGAACCA | 126 bp |
|  | R：AGGCCAATACCCTACCGTCT |  |
|  | P：TGACATATCATTCAAGTTTCTGAC |  |
|  |  |  |
| 18S rRNA nested PCR | Primary | 1307 bp |
|  | F1：TTCTAGAGCTAATACATGCG |  |
|  | R1：CCCATTTCCTTCGAAACAGGA |  |
|  |  |  |
|  | Secondary | 820 bp |
|  | F2：GGAAGGGTTGTATTTATTAGATAAAG |  |
|  | R2：AAGGAGTAAGGAACAACCTCCA |  |
|  |  |  |
| COWP qPCR | Cryptosporidium | 151 bp |
|  | F：CAAATTGATACCGTTTGTCCTTCTG |  |
|  | R：GGCATGTCGATTCTAATTCAGCT |  |
|  | NED-ATTCAATTTGTCAGGACAACAATGTATGGCA-MGB |  |
|  |  |  |
|  | C. baileyi | 176 bp |
|  | F：GGAACTTGTAAACTCATCCAACA |  |
|  | R：GGTGGACATGTTGATTCTAATTCAGTT |  |
|  | FAM-CATCTGGATTTGTTGAAGAAGG-MGB |  |

Supplemental Table S4 The sequencing results of COI PCR amplicon of birds and 18S rRNA PCR amplicon of *Cryptosporidium*.

| Target | Accession number | Sequence (5’-3’) |
| --- | --- | --- |
| Bird COI | PV124901 | TAGAAGCTGGCGCCGGCACAGGCTGAACTGTCTACCCTCCCCTAGCAGGTAACCTCGCCCACGCCGGAGCTTCAGTAGACCTGGCTATCTTCTCACTCCACTTAGCCGGTATCTCCTCCATCCTTGGAGCCATCAACTTTATCACCACAGCCATCAACATAAAACCCCCCGCACTCTCACAATACCAAACCCCACTATTTGTCTGATCCGTACTAATTACCGCCATCCTACTCCTTCTATCACTCCCCGTACTCGCCGCCGGTATTACAATATTACTAACTGACCGAAACCTAAACACCACATTCTTCGACCCCGCTGGAGGAGGAGACCCAATCCTGTACCAACACCTATTCTGATTCTTCGGCCACCCAG |
|  | PV124902 | GCGCCGGCACAGGCTGAACTGTCTACCCTCCCCTAGCAGGTAACCTTGCCCACGCCGGAGCTTCAGTAGACCTGGCTATCTTCTCACTCCACTTAGCCGGTATCTCCTCCATCCTTGGAGCCATCAACTTTATCACCACAGCCATCAACATAAAACCCCCCGCACTCTCACAATACCAAACCCCACTATTTGTCTGATCCGTACTAATTACCGCCATCCTACTCCTTCTATCACTCCCCGTACTCGCCGCCGGTATTACAATATTACTAACTGACCGAAACCTAAACACCACATTCTTCGACCCCGCTGGAGGAGGAGACCCAATCCTGTACCAACACCTATTCTGATTCTTGGGCCACCCAG |
|  | PV124903 | GTATACCCCCCTCTAGCTGGCAATCTAGCCCATGCTGGAGCTTCAGTAGACCTAGCAATCTTCTCCCTTCACTTAGCAGGTGTATCCTCCATTCTAGGTGCTATTAACTTTATCACTACAGCCATCAACATAAAACCTCCCGCTCTCTCACAATATCAAACCCCACTATTCGTATGATCCGTACTCATTACTGCCGTACTATTACTACTCTCACTTCCAGTGCTTGCTGCAGGCATCACTATACTACTTACAGACCGAAACCTAAACACAACATTCTTCGATCCCGCCGGAGGCGGTGACCCTGTACTGTATCAACACCTCTTCTGATTCTTCGGCCACCCAG |
|  | PV124904 | TGGTGTTTAGGTTTCGGTCAGTTAGTAATATTGTAATACCGGCGGCGAGTACGGGGAGTGATAGAAGGAGTAGGATGGCGGTAATTAGTACGGATCAGACAAATAGTGGGGTTTGGTATTGTGAGAGTGCGGGGGGTTTTATGTTGATGGCTGTGGTGATAAAGTTGATGGCTCCAAGGATGGAGGAGATACCGGCTAAGTGGAGTGAGAAGATAGCCAGGTCTACTGAAGCTCCGGCGTGGGCGAGGTTACCTGCTAGGGGAGGGTAGACAGTTCAGCCTGTGCCGGCGCCAGCTTCTACAGTGGATGAGGCTAGCAGTAGGAGGAATGATGGGGGGAGGAGTC |
|  | PV124905 | GTAGAAGCAGGTGCAGGTACAGGATGAACAGTATATCCCCCACTTGCTGGCAACCTAGCCCATGCCGGAGCTTCCGTAGACCTAGCTATCTTCTCTCTCCACTTAGCAGGTGTCTCCTCCATCCTAGGTGCTATTAATTTCATCACAACTGCTATTAACATAAAACCCCCAGCCCTCTCCCAATACCAAACACCCCTATTCGTATGATCAGTACTCATTACCGCTGTCTTACTATTACTCTCTCTCCCAGTCCTCGCTGCTGGTATTACTATGCTACTAACAGACCGAAACCTAAATACTACATTCTTCGACCCCGCCGGAGGAGGAGACCCAGTTCTATATCAACATCTCTTCTGATTCT |
|  | PV124906 | ACAGTAGAAGCAGGTGCAGGTACAGGATGAACAGTATATCCTCCACTTGCTGGCAACCTAGCCCATGCCGGAGCTTCCGTAGACCTAGCTATCTTCTCTCTCCACTTAGCAGGTGTCTCCTCCATCCTAGGTGCTATTAATTTCATCACAACTGCTATTAACATAAAACCCCCAGCCCTCTCCCAATACCAAACACCCCTATTCGTATGATCAGTACTCATTACCGCTGTCTTACTATTACTCTCTCTCCCAGTCCTCGCTGCTGGTATTACTATGCTACTAACAGACCGAAACCTAAATACTACATTCTTCGACCCCGCCGGAGGAGGAGACCCAGTTCTATATCAACATCTCTTCTGATTC |
|  | PV124907 | CGGCACAGGATGAACAGTATACCCCCCTCTAGCTGGCAATCTAGCCCATGCTGGAGCCTCAGTAGACCTAGCAATCTTCTCTCTTCACTTAGCAGGTGTGTCTTCCATTCTGGGTGCTATCAACTTTATCACTACAGCCATCAACATAAAACCCCCTGCCCTCTCACAATATCAAACCCCACTATTCGTATGATCCGTACTCATCACTGCCGTCCTATTACTACTTTCACTCCCAGTGCTTGCCGCAGGCATTACTATGCTACTTACAGACCGAAACCTAAACACAACATTCTTCGATCCCGCCGGAGGCGGTGACCCTGTACTGTACCAACACCTCTTCTGATT |
|  | PV124908 | GCTGGCGCCGGCACAGGCTGAACTGTCTACCCTCCCCTAGCAGGTAACCTCGCCCACGCCGGAGCTTCAGTAGACCTGGCTATCTTCTCACTCCACTTAGCCGGTATCTCCTCCATCCTTGGGGCCATCAACTTTATTACCACAGCTATCAACATAAAACCCCCCGCACTCTCACAATACCAAACCCCACTATTTGTCTGATCCGTACTAATTACCGCCATCCTACTCCTTCTATCGCTCCCCGTACTCGCCGCCGGTATTACAATACTACTAACTGACCGAAACCTAAACACCACATTCTTCGATCCCGCTGGAGGAGGAGACCCAATCCTGTACCAACACCTATTCTGATTCTCGGACCACCCAG |
|  | PV124909 | TGTACCCGCCCCTAGCAGGCAACCTGGCCCACGCCGGGGCCTCAGTAGACCTGGCCATCTTCTCACTCCACCTAGCCGGTGTCTCCTCCATCCTCGGAGCCATTAACTTCATCACCACAGCCATTAACATAAAACCCCCCGCACTCTCACAATATCAAACACCACTCTTCGTCTGATCGGTCCTAATTACCGCCATCCTGCTCCTCCTATCACTCCCCGTCCTCGCCGCCGGCATCACAATGCTATTAACCGACCGAAACCTAAACACCACATTCTTTGACCCCGCCGGAGGAGGAGATCCAATCCTATACCAGCACCTATTTTGATTT |
|  | PV124910 | TGTTGTGTTTAGGTTTCGGTCTGTTAATAGCATAGTGATGCCGGCGGCGAGTACTGGGAGTGAGAGTAGTAGAAGGACGGCAGTGATAAGTACGGATCACACAAATAGGGGGGTTTGGTATTGTGAAAGGGCAGGAGGTTTTATGTTAATAGCCGTAGTGATAAAGTTGATAGCACCTAGGATGGAGGAGACACCTGCCAGGTGGAGAGAGAAGATTGCTAAATCTACTGAAGCTCCAGCATGGGCTAGATTGCCAGCTAGGGGAGGGTATACAGTTCATCCTGTACCTGCTCCAGCCTCTACTGTGGAGGAGGCTAGGAGGAGTAAGAATGATGGAGGGAGTAGTCAGA |
|  | PV124911 | TGTTGTGTTTAGGTTTCGATCTGTTAGTAACATGGTAATGCCAGCGGCGAGTACTGGGAGTGAGAGTAGTAGTAGGACAGCAGTGATGAGTACGGATCATACGAATAGAGGGGTTTGATATTGTGAAAGGGCAGGGGGTTTTATGTTGATGGCTGTGGTGATGAAATTAATAGCACCTAGGATAGAGGATACACCTGCTAGATGAAGGGAGAAGATTGCTAAGTCTACTGAAGCTCCAGCATGAGCTAGATTGCCAGCTAGGGGGGGATATACAGTTCATCCTGTGCCTACTCCAGCTTCTACTGTGGAAGAGGCTAGGAGGAGTAAGAATGATGGGGGTAGCAGTCAGA |
|  | PV124912 | AAGCTGGCGCCGGCACAGGCTGAACTGTCTACCCTCCCCTAGCAGGTAACCTCGCCCACGCCGGAGCTTCAGTAGACCTGGCTATCTTCTCACTCCACTTAGCCGGTATCTCCTCCATCCTTGGAGCCATCAACTTTATCACCACAGCCATCAACATAAAACCCCCCGCACTCTCACAATACCAAACCCCACTATTTGTCTGATCCGTACTAATTACCGCCATCCTACTCCTTCTATCACTCCCCGTACTCGCCGCCGGTATTACAATATTACTAACTGACCGAAACCTAAACACCACATTCTTCGACCCCGCTGGAGGAGGAGACCCAATCCTGTACCAACACCT |
|  | PV124913 | GGAGCAGGAACAGGATGAACTGTATATCCCCCCTTAGCTGGCAATTTAGCCCACGCCGGAGCTTCAGTAGACCTAGCCATCTTCTCCCTCCACTTAGCAGGTGTATCCTCCATCCTAGGCGCAATCAACTTCATCACAACTGCTATCAACATAAAACCACCCGCCCTTTCACAATACCAAACCCCTCTATTCGTTTGATCTGTCCTCATCACCGCCGTCTTATTACTCCTATCACTCCCAGTCCTAGCCGCTGGCATTACCATACTACTAACAGACCGAAACCTAAACACCACATTCTTTGACCCCGCTGGAGGAGGTGACCCAGTCCTATACCAACACCTCTTCTGATTT |
|  | PV124914 | GAAGCCGGAGCCGGTACAGGGTGAACAGTATACCCCCCTCTAGCTGGCAATCTAGCCCATGCTGGAGCTTCAGTAGACCTAGCAATCTTCTCCCTTCACTTAGCAGGTGTATCCTCCATTCTAGGTGCTATTAACTTTATCACTACAGCCATCAACATAAAACCTCCCGCTCTCTCACAATATCAAACCCCACTATTCGTATGATCCGTACTCATTACTGCCGTACTATTACTACTCTCACTTCCAGTGCTTGCTGCAGGCATCACTATACTACTTACAGACCGAAACCTAAACACAACATTCTTCGATCCCGCCGGAGGCGGTGACCCTGTACTGTATCAACACCTCTTCTGAT |
|  | PV124915 | CCGGAGCTGGTACAGGATGAACAGTATACCCCCCTCTCGCTGGTAATCTAGCCCATGCTGGTGCCTCAGTAGACCTGGCCATCTTCTCTCTCCACCTAGCAGGGGTCTCCTCTATTCTTGGTGCCATCAATTTCATCACAACTGCCATTAACATAAAACCCCCAGCCCTCTCCCAATATCAAACTCCCTTGTTCGTATGATCAGTACTTATTACCGCCGTCCTTCTTTTACTCTCCCTCCCAGTCCTTGCCGCCGGCATCACTATACTATTAACAGATCGAAACCTAAATACCACATTCTTTGATCCCGCTGGAGGAGGAGACCCAGTCCTATACCAACACCTTTTCTG |
|  | PV124916 | AGCGGGGGCAGGCACAGGATGAACTGTATACCCTCCTCTAGCTGGTAATCTAGCTCATGCTGGAGCTTCAGTAGATTTAGCAATCTTCTCCCTCCATCTAGCAGGTGTATCATCCATCCTGGGCGCTATCAACTTTATCACTACAGCTATCAACATAAAACCCCCTGCTCTTTCACAATACCAGACCCCTCTATTCGTATGATCCGTACTTATTACTGCTGTCCTATTACTACTCTCGCTCCCAGTACTTGCCGCCGGCATCACTATGCTATTAACAGACCGAAACCTAAACACAACATTCTTTGACCCTGCCGGAGGTGGTGACCCCGTACTATACCAACATCTCTTCTGATTC |
|  | PV124917 | GAGGCTGGAGCAGGTACAGGATGAACTGTATACCCTCCCCTAGCTGGCAATCTAGCCCATGCTGGAGCTTCAGTAGATTTAGCAATCTTCTCTCTCCACCTGGCAGGTGTCTCCTCCATCCTAGGTGCTATCAACTTTATCACTACGGCTATTAACATAAAACCTCCTGCCCTTTCACAATACCAAACCCCCCTATTTGTGTGATCCGTACTTATCACTGCCGTCCTTCTACTACTCTCACTCCCAGTACTCGCCGCCGGCATCACTATGCTATTAACAGACCGAAACCTAAACACAACATTCTTTGATCCTGCCGGAGGTGGTGACCCTGTACTATATCAACATCTATTTTGAT |
|  | PV124918 | TGTTGTGTTTAGGTTTCGGTCTGTAAGTAGCATAGTAATGCCTGCGGCAAGTACTGGGAGTGAAAGTAGTAATAGGACGGCAGTGATGAGTACGGATCATACGAATAGTGGGGTTTGATATTGTGAAAGGGCAGGGGGTTTTATGTTGATGGCTGTAGTGATAAAGTTAATAGCACCCAGGATGGAAGACACACCCGCTAAGTGAAGAGAGAAGATTGCTAGGTCTACTGAGGCTCCAGCATGGGCTAGATTGCCAGCTAGAGGGGGGTATACTGTTCATCCTGTACCGGCTCCAGCTTCTACTGTGGAAGAGGCTAGGAGGAGTAGGAATGATGGAGGTAACAGT |
|  | PV124919 | TACCCTCCCCTAGCAGGTAACCTAGCCCACGCCGGAGCTTCAGTAGACCTGGCTATCTTCTCACTCCACTTAGCCGGTATCTCCTCCATCCTTGGAGCCATCAACTTTATCACCACAGCCATCAACATAAAACCCCCCGCACTCTCACAATACCAAACCCCACTATTCGTCTGATCCGTACTAATTACCGCCATCCTACTCCTTCTATCACTCCCCGTACTCGCCGCCGGTATTACAATACTACTAACTGACCGAAACCTAAACACCACATTCTTCGACCCCGCTGGAGGAGGAGACCCAATCCTGTACCAACACCTATTCTGATTCTTCGGC |
|  | PV124920 | ACCCACCCCTAGCAGGGAACTACTCCCACCCTGGAGCTTCCGTAGACCTGGCCATTTTCTCACTTCACTTAGCTGGTGTCTCCTCCATCCTTGGAGCCATCAACTTCATTACCACAGTCATCAACATAAAACCCCCCGCACTCTCACAATACCAAACTCCACTATTCGTCTGATCCGTCCTAATTACCGCCATCCTACTCCTTCTATCACTCCCTGTGCTAGCCGCTGGTATCACAATGCTATTAACCGACCGAAACCTAAACACCACCTTCTTCGACCCCGCCGGAGGAGGAGACCCAATCCTGTATCAACACTTATTCTGATTTTTCGGCCACCCA |
|  | PV124921 | GTAGAAGCTGGCGCCGGCACAGGCTGAACTGTCTACCCTCCCCTAGCAGGTAACCTTGCCCACGCCGGAGCTTCAGTAGACCTGGCTATCTTCTCACTCCACTTAGCCGGTATCTCCTCCATTCTTGGGGCCATCAACTTTATTACCACAGCTATCAACATAAAACCCCCCGCACTCTCACAATACCAAACCCCACTATTTGTCTGATCCGTACTAATTACCGCCATCCTACTCCTTCTATCACTCCCCGTACTCGCCGCCGGTATTACAATACTACTAACTGATCGAAACCTAAACACCACATTCTTCGATCCCGCTGGAGGGGGAGACCCAATCCTGTACCAGCACCTATTCTGATTCTC |
|  | PV124922 | GCGCCGGCACAGGCTGAACTGTCTACCCTCCCCTAGCAGGTAACCTCGCCCACGCCGGAGCTTCAGTAGACCTGGCTATCTTCTCACTCCACTTAGCCGGTATCTCCTCCATTCTTGGAGCCATCAACTTTATTACCACAGCTATCAACATAAAACCCCCCGCACTCTCACAATACCAAACCCCACTATTTGTCTGATCCGTGCTAATTACCGCCATCCTACTCCTTCTATCACTCCCCGTACTCGCCGCCGGTATTACAATACTACTAACTGATCGAAACCTAAACACCACATTCTTCGATCCCGCTGGAGGAGGAGACCCAATCCTGTACCAACACCTATTCTGATTCTC |
|  | PV124923 | GTAGAAGCTGGCGCCGGCACAGGCTGAACTGTCTACCCTCCCCTAGCAGGTAACCTTGCCCACGCCGGAGCTTCAGTAGACCTGGCTATCTTCTCACTCCACTTAGCCGGTATCTCCTCCATCCTTGGAGCCATCAACTTTATCACCACAGCCATCAACATAAAACCCCCCGCACTCTCACAATACCAAACCCCACTATTTGTCTGATCCGTGCTAATTACCGCCATCCTACTCCTTCTATCACTCCCCGTACTCGCCGCCGGTATTACAATATTACTAACTGATCGAAACCTAAACACCACATTCTTCGACCCCGCTGGAGGAGGAGACCCAATCCTGTACCAACACCTATTCTGATTCTTCGGCCACCCAG |
|  | PV124924 | AACTGTCTACCCTCCCCTAGCAGGTGACCTTGCCCACGCCGGAGCTTCAGTAGACCTGGCTATCTTCTCACTCCACTTAGCCGGTATCTCCTCCATTCTTGGAGCCATCAACTTTATTACCACAGCTATCAACATAAAACCCCCCGCACTCTCACAATACCAAACCCCACTATTTGTCTGATCCGTACTAATTACCGCCATCCTACTCCTTCTATCACTCCCCGTACTCGCCGCCGGTATTACAATATTACTAACTGATCGAAACCTAAACACCACATTCTTCGACCCCGCTGGAGGAGGAGACCCAATCCTGTACCAACACCTATTCTGATTCTTCGGACACCCA |
|  | PV124925 | GTAGAAGCTGGCGCCGGCACAGGCTGAACTGTCTACCCTCCCCTAGCAGGTAACCTCGCCCACGCCGGAGCTTCAGTAGACCTGGCCATCTTCTCACTCCACTTAGCCGGTATCTCCTCCATTCTTGGGGCCATCAACTTTATTACCACAGCTATCAACATAAAACCCCCCGCACTCTCACAATACCAAACCCCACTATTTGTCTGATCCGTACTAATTACCGCCATCCTACTCCTTCTATCACTCCCCGTACTCGCCGCCGGTATTACAATACTACTAACTGATCGAAACCTAAACACCACATTCTTCGATCCCGCTGGAGGGGGAGACCCAATCCTGTACCAGCACCTATTCTGATTCTTCGGCCACCCA |
|  | PV124926 | GAGCCGGTACAGGGTGAACTGTATACCCTCCCCTAGCTGGCAACCTCGCCCATGCCGGAGCTTCAGTAGACCTGGCTATCTTCTCCCTTCACTTAGCCGGTGTCTCCTCCATTCTAGGGGCTATTAACTTTATCACTACAGCCATCAACATAAAACCCCCCGCTCTCTCACAATATCAAACCCCACTATTCGTATGATCCGTACTCATTACTGCCGTCCTATTACTACTATCACTTCCCGTGCTCGCTGCCGGCATCACTATACTACTTACTGACCGAAACCTAAACACCACATTCTTCGATCCCGCCGGAGGGGGAGACCCTGTCCTGTATCAACACCTATTCTGATTCTTCGGACACCCA |
|  | PV124927 | CTCCATCCTGGGAGCTATTAACTTTATCACCACAGCCATCAACATAAAACCCCCCGCACTCTCACAATACCAAACCCCACTCTTTGTCTGATCCGTCCTAATCACCGCCATTCTGCTCCTTCTATCACTACCTGTTCTCGCCGCTGGCATCACAATACTACTAACCGACCGAAACCTAAACACCACATTCTTTGACCCCGCCGGAGGAGGTGACCCAATCCTGTACCAACACCTATTCTGATTCTTCGGC |
|  | PV124928 | TAGAAGCAGGAGCAGGTACAGGATGAACAGTCTACCCACCACTAGCTGGTAACCTAGCCCACGCCGGAGCTTCAGTAGACCTAGCCATCTTCTCTCTTCACCTAGCAGGTGTATCTTCCATCCTGGGGGCAATCAATTTCATCACAACAGCCATCAACATAAAACCACCAGCCCTATCACAATACCAAACACCCTTATTCGTGTGATCCGTCCTAATTACCGCTGTCCTATTACTCCTCTCTCTCCCAGTCCTTGCCGCTGGCATCACCATACTACTAACAGACCGAAATCTCAATACTACATTCTTCGACCCTGCTGGAGGAGGAGACCCTGTCCTATATCAACATCTCTTCTGATTCTTCGGCC |
|  | PV124929 | TAGAAGCCGGAGCAGGTACAGGATGAACGGTGTATCCCCCACTCGCCGGCAACCTGGCCCACGCTGGAGCCTCCGTCGACCTTGCCATCTTCTCCCTCCACCTGGCAGGGGTCTCCTCTATTCTAGGTGCCATCAACTTCATCACAACTGCCATTAACATAAAACCTCCAGCCCTCTCCCAATACCAAACACCTCTGTTTGTATGATCAGTACTTATTACTGCTGTCCTACTCTTACTTTCTCTTCCAGTTCTTGCTGCTGGTATTACTATGCTACTAACAGACCGAAACCTTAACACCACATTCTTCGATCCTGCTGGAGGAGGAGATCCAGTTCTATACCAACATCTC |
|  | PV124930 | TCTAGCTGGCAATCTAGCCCATGCTGGAGCCTCAGTAGACCTGGCAATCTTCTCTCTTCACTTAGCAGGTGTGTCTTCCATCCTGGGTGCTATTAACTTTATCACTACAGCTATCAACATAAAACCCCCTGCCCTCTCACAATATCAAACCCCCCTATTCGTATGATCCGTACTCATCACTGCCGTCCTATTACTACTTTCACTTCCAGTGCTTGCCGCAGGCATTACTATGCTACTTACAGACCGAAACCTAAACACAACATTCTTCGATCCCGCCGGAGGTGGTGACCCTGTACTGTATCAACACCTCTTCTGATTCTTCGGCCACCCAG |
|  | PV124931 | TACCCCCCTCTAGCTGGTAACCTAGCTCACGCCGGAGCCTCAGTAGACCTGGCTATTTTTTCCCTCCACCTAGCAGGTGTATCCTCAATCCTAGGTGCAATCAACTTCATCACAACCGCCATCAATATAAAACCTCCTGCCCTATCACAATACCAAACTCCCCTATTTGTATGATCCGTACTCATCACTGCCGTCCTACTGCTCCTTTCACTCCCAGTTCTTGCTGCTGGCATCACCATACTATTAACAGACCGAAACCTAAACACCACATTCTTCGACCCTGCCGGAGGCGGAGACCCAGTCCTATATCAACACCTCTTCTGATTCTTCGGCCACCCA |
|  | PV124932 | AGCTGGCAACCTAGCTCACGCCGGAGCCTCAGTTGACCTAGCTATTTTTTCCCTCCACCTAGCAGGTGTATCATCAATCCTAGGTGCAATCAACTTCATCACAACCGCCATCAACATAAAACCCCCTGCCCTATCACAATACCAAACTCCCCTATTTGTATGATCCGTCCTCATCACCGCCGTCCTATTACTCCTCTCACTCCCAGTTCTCGCTGCTGGCATCACAATGCTGCTAACAGACCGAAACCTAAACACCACATTCTTCGACCCCGCCGGAGGCGGAGATCCAGTCCTATATCAACACCTCTTCTGATTCTTCGGCCACCCAG |
|  | PV124933 | CTAGCTGGCAATCTAGCCCATGCTGGAGCTTCAGTAGACCTGGCAATCTTCTCCCTTCACTTAGCAGGTGTATCCTCCATTCTAGGTGCTATTAACTTTATCACTACTGCCATCAACATAAAACCTCCCGCTCTCTCACAATATCAAACCCCACTATTCGTATGATCCGTACTCATTACTGCCGTACTATTACTACTCTCACTTCCAGTGCTTGCTGCAGGCATCACTATACTACTTACAGACCGAAACCTAAACACAACATTCTTCGATCCCGCCGGAGGAGGTGACCCTGTACTGTATCAACACCTCTTCTGATTCT |
|  | PV124934 | AGCAGGAACAGGATGAACTGTATATCCCCCCTTAGCTGGCAATTTAGCCCACGCCGGAGCTTCAGTAGACCTAGCCATCTTCTCCCTCCACTTAGCAGGTGTATCCTCCATCCTAGGCGCAATCAACTTCATCACAACTGCTATCAACATAAAACCACCCGCCCTTTCACAATACCAAACCCCTCTATTCGTTTGATCTGTCCTCATCACCGCCGTCTTATTACTCCTATCACTCCCAGTCCTAGCCGCTGGCATTACCATACTACTAACAGACCGAAACCTAAACACCACATTCTTTGACCCCGCTGGAGGAGGTGACCCAGTCCTATACCAACACCTCTTCTG |
|  | PV124935 | AGAAGCCGGAGCAGGCACAGGATGAACCGTATACCCCCCTCTAGCTGGTAACCTAGCTCACGCCGGAGCCTCAGTAGACCTGGCTATTTTTTCCCTCCACCTAGCAGGTGTATCCTCAATCCTAGGTGCAATCAACTTCATCACAACCGCCATCAATATAAAACCTCCTGCCCTATCACAATACCAAACTCCCCTATTTGTATGATCCGTACTCATCACTGCCGTCCTACTGCTCCTTTCACTCCCAGTTCTTGCTGCTGGCATCACCATACTATTAACAGACCGAAACCTAAACACCACATTCTTCGACCCTGCCGGAGGCGGAGACCCAGTCCTATATCAACACCTCTTCTGATTCT |
|  | PV124936 | TCCACAGTAGAAGCAGGAGCAGGCACAGGATGAACAGTTTACCCCCCACTAGCCGGCAACCTAGCACATGCAGGCGCTTCAGTTGACCTAGCCATCTTCTCCCTCCACTTAGCAGGTGTCTCATCTATTCTAGGCGCCATCAATTTTATTACAACTGCCATCAACATAAAACCACCCGCCCTATCCCAATATCAAACTCCTCTGTTCGTATGATCTGTCCTCATTACCGCTGTTCTACTACTGCTATCCCTCCCTGTCCTTGCCGCTGGCATTACCATACTACTAACCGACCGAAACCTAAATACCACATTCTTCGACCCAGCCGGAGGAGGTGATCCCATCCTATACCAACACCTCTTCTGATTT |
| Cryptosporidium 18S rRNA | PQ047601 | AATATGTGACATATCATTCAAGTTTCTGACCTATCAGCTTTAGACGGTAGGGTATTGGCCTACCGTGGCTATGACGGGTAACGGGGAATTAGGGTTCGATTCCGGAGAGGGAGCCTGAGAAACGGCTACCACATCTAAGGAAGGCAGCAGGCGCGCAAATTACCCAATCCTAATACAGGGAGGTAGTGACAAGAAATAACAATACAGAACCTTACGGTTTTGTAATTGGAATGAGTTAAGTATAAACCCCTTAACAAGTATCAATTGGAGGGCAAGTCTGGTGCCAGCAGCCGCGGTAATTCCAGCTCCAATAGCGTATATTAAAGTTGTTGCAGTTAAAAAGCTCGTAGTTGGATTTCTGCTGATTTTTATATATAATACTACGGTATTTATATAAAATCAGCATAATCCGCATTACTTAGAGTATGCGGAACTTTACTTTGAGAAAATTAGAGTGCTTAAAGCAGGCAATTGCCTTGAATACTCCAGCATGGAATAATATTAAGGATTTTTATCCTTTTTATTGGTTCTAGGATAAAAATAATGATTAATAGGGACAGTTGGGGGCATTTGTATTTAACAGTCAGAGGTGAAATTCTTAGATTTGTTAAAGACAAACTACTGCGAAAGCATTTGCCAAGGATGTTTTCATTAATCAAGAACGAAAGTTAGGGGATCGAAGACGATCAGATACCGTCGTAGTCTTAACCATAAACTATGCCGACTAGAGATTGGAGTG |
|  | PQ047618 | CGATTCCGGAGAGGGAGCCTGAGAAACGGCTACCACATCTAAGGAAGGCAGCAGGCGCGCAAATTACCCAATCCTAATACAGGGAGGTAGTGACAAGAAATAACAATACAGAACTTTACGGTTTTGTAATTGGAATGAGTTAAGTATAAACCCCTTAACAAGTATCAATTGGAGGGCAAGTCTGGTGCCAGCAGCCGCGGTAATTCCAGCTCCAATAGCGTATATTAAAGTTGTTGCAGTTAAAAAGCTCGTAGTTGGATTTCTGCTAATTTTTGCATATAATACCACGGTATTTATGTAAAATTAGCATAATCCGCATTACCTTGCGTATGCGGAATTTTACTTTGAGAAAATTAGAGTGCTTAAAGCAGGCAATTGCCTTGAATACTCCAGCATGGAATAATATTAAGGATTTTTATCCTTTTTATTGGTTCTAGGATAAAAATAATGATTAATAGGGACAGTTGGGGGCATTTGTATTTAACAGTCAGAGGTGAAATTCTTAGATTTGTTAAAGACAAACTACTGCGAAAGCATTTGCCAAGGATGTTTTCATTAATCAAGAACGAAAGTTAGGGGATCGAAGACGATCAGATACCGTCGTAGTCTTAACCATAAACTATGCCGACTAAGATGAT |
|  | PQ047619 | TGAGAAACGGCTACCACATCTAAGGAAGGCAGCAGGCGCGCAAATTACCCAATCCTAATACAGGGAGGTAGTGACAAGAAATAACAATACAGAACTTTACGGTTTTGTAATTGGAATGAGTTAAGTATAAACCCCTTAACAAGAATCAATTGGAGGGCAAGTCTGGGGCCAGCAGCCGCGGGAATTCCAGCTCCAATAGCGTATATTAAAGTTGTTGCAGTTAAAAAGCTCGTAGATGGATTTCTGCTAATTTTTGCATATAATACCACGGTATTTATGTAAAATTAGCATAATCCGCATTACCTTGCGTATGCGGAATTTTACTTTGAGAAAATTAGAGTGCTTAAAGCAGGCAATTGCCTTGAATACTCCAGCATGGAATAATATTAAGGATTTTTATCCTTTTTATTGGTTCTAGGATAAAAATAATGATTAATAGGGACAGTTGGGGGCATTTGTATTTAACAGTCAGAGGTGAAATTCTTAGATTTGTTAAAGACAAACTACTGCGAAAGCATTTGCCAAGGATGTTTTCATTAATCAAGAACGAAAGTTAGGGGATCGAAGACGATCAGATACCGTCGTAGTCTTATCCATATACTATGTCGACTAGAGATGGAGG |
|  | PQ047620 | ATTCCGGAGAGGGAGCCTGAGAAACGGCTACCACATCTAAGGAAGGCAGCAGGCGCGCAAATTACCCAATCCTGACACAGGGAGGTAGTGACAAGAAATAACAATACAGGGCCTAACGGTCTTGTAATTGGAATGAGTTAAGTATAAACCCCTTTACAAGTATCAATTGGAGGGCAAGTCTGGTGCCAGCAGCCGCGGTAATTCCAGCTCCAATAGCGTATATTAAAGTTGTTGCAGTTAAAAAGCTCGTAGTTGGATTTCTGTTAATTCTTATATATAATACTACGGTATTTATATAACATTAACATAATTCATATTACTTATTTAAAGTATATGAAATTTTACTTTGAGAAAATTAGAGTGCTTAAAGCAGGCTATTGCCTTGAATACTCCAGCATGGAATAATATTAAAGATTTTTATCTTTCTTATTGGTTCTAAGATAAAAATGATGATTAATAGGGACAGTTGGGGGCATTTGTATTTAACAGTCAGAGGTGAAATTCTTAGATTTGTTAAAGACAAACTACTGCGAAAGCATTTGCCAAGGATGTTTTCATTAATCAAGAACGAAAGTTAGGGGATCGAAGACGATCAGATACCGTCGTAGTCTTAACCATAAACTATGCCGACTAGA |
|  | PQ047621 | GGGTTCGATTCCGGAGAGGGAGCCTGAGAAACGGCTACCACATCCAAGGAAGGCAGCAGGCGCGCAAATTACCCAATCCTAATACAGGGAGGTAGTGACAAGAAATAACAATACAGAACTTTACGGTTTTGTAATTGGAATGAGTTAAGTATAAACCCCTTAACAAGTATCAATTGGAGGGCAAGTCTGGGGCCAGCAGCCGCGGGAATTCCAGCTCCAATAGCGTATATTAAAGTTGTTGCAGTTAAAAAGCTCGTAGTTGGATTTCTGCTAATTTTTACATACAATACTACGGTATTTATGTAAAATTAGCATAATCCGCATTACCTCGCGTATGCGGAACTTTACTTTGAGAAAATTAGAGTGCTTAAAGCAGGCGATTGCCTTGAATACTCCAGCATGGAATAATATTAAGGATTTTTATCCTTTTTATTGGTTCTAGGATAAAAATAATGATTAATAGGGACAGTTGGGGGCATTTGTATTTAACAGTCAGAGGTGAAATTCTTAGATTTGTTAAAGACAAACTACTGCGAAAGCATTTGCCAAGGATGTTTTCATTAATCAAGAACGAAAGTTAGGGGATCGAAGACGATCAGATACCGTCGTAGTCTTAACCATAAACTATGCCGACTAGAGATTTGGAGGGTG |
|  | PQ047622 | AGGGAGCCTGAGAAACGGCTACCACATCTAAGGAAGGCAGCAGGCGCGCAAATTACCCAATCCTAATACAGGGAGGTAGTGACAAGAAATAACAATACAGAACTTTACGGTTTTGTAATTGGAATGAGTTAAGTATAAACCCCTTAACAAGTATCAATTGGAGGGCAAGTCTGGTGCCAGCAGCCGCGGTAATTCCAGCTCCAATAGCGTATATTAAAGTTGTTGCAGTTAAAAAGCTCGTAGTTGGATTTCTGCTAATTTTTGCATACAATACCACGGTATTTATGTAAAATTAGCATAATCCGCATTACCTTGCGTATGCGGAATTTTACTTTGAGAAAATTAGAGTGCTTAAAGCAGGCAATTGCCTTGAATACTCCAGCATGGAATAATATTAAGGATTTTTATCCTTTTTATTGGTTCTAGGATAAAAATAATGATTAATAGGGACAGTTGGGGGCATTTGTATTTAACAGTCAGAGGTGAAATTCTTAGATTTGTTAAAGACAAACTACTGCGAAAGCATTTGCCAAGGATGTTTTCATTAATCAAGAACGAAAGTTAGGGGATCGAAGACGATCAGATACCGTCGTAGTCTTAACCATAAACTATGCCGACTAGAGATTGGAGTT |
|  | PQ047623 | AGGGAGCCTGAGAAACGGCTACCACATCTAAGGAAGGCAGCAGGCGCGCAAATTACCCAATCCTAATACAGGGAGGTAGTGACAAGAAATAACAATACAGAACTTTACGGTTTTGTAATTGGAATGAGTTAAGTATAAACCCCTTAACAAGTATCAATTGGAGGGCAAGTCTGGTGCCAGCAGCCGCGGTAATTCCAGCTCCAATAGCGTATATTAAAGTTGTTGCAGTTAAAAAGCTCGTAGTTGGATTTCTGCTAATTTTTGCATACAATACCACGGTATTTATGTAAAATTAGCATAATCCGCATTACCTTGCGTATGCGGAATTTTACTTTGAGAAAATTAGAGTGCTTAAAGCAGGCAATTGCCTTGAATACTCCAGCATGGAATAATATTAAGGATTTTTATCCTTTTTATTGGTTCTAGGATAAAAATAATGATTAATAGGGACAGTTGGGGGCATTTGTATTTAACAGTCAGAGGTGAAATTCTTAGATTTGTTAAAGACAAACTACTGCGAAAGCATTTGCCAAGGATGTTTTCATTAATCAAGAACGAAAGTTAGGGGATCGAAGACGATCAGATACCGTCGTAGTCTTAACCATAAACTATGCCGACTAGAGATTGGAGGTTG |
|  | PQ047624 | ACGCGACATATCATTCAAGTTTCTGACCTATCAGCTTTAGACGGTAGGGTATTGGCCTACCGTGGCTATGACGGGTAACGGGGAATTAGGGTTCGATTCCGGAGAGGGAGCCTGAGAAACGGCTACCACATCTAAGGAAGGCAGCAGGCGCGCAAATTACCCAATCCTGACACAGGGAGGTAGTGACAAGAAATAACAATACAGGGCCTAACGGTCTTGTAATTGGAATGAGTGAAGTATAAACCCCTTTACGAGTATCAATTGGAGGGCAAGTCTGGTGCCAGCAGCCGCGGTAATTCCAGCTCCAATAGCGTATATTAAAGTTGTTGCAGTTAAAAAGCTCGTAGTTGGATTTCTGTTGTATTTTTATAATATTACTAAGGTAACATTATAATAACAACATCCTTCCCAATATATTTTTTAATATATGGGAAATTTTACTTTGAGAAAATTAGAGTGCTTAAAGCAGGCAACTGCCTTGAATACTCCAGCATGGAATAATAAGTAAGGACTTTTGTCTTTCTTGTTGGTTCTAGGATAAAAGTAATGGTTAATAGGGACAGTTGGGGGCATTCGTATTTAACAGCCAGAGGTGAAATTCTTAGATTTGTTAAAGACGAACTACTGCGAAAGCATTTGCCAAGGATGTTTTCATTAATCAAGAACGAAAGTTAGGGGATCGAAGACGATCAGATACCGTCGTAGTCTTAACCATAAACTATGCCGACTAGAGATGAGTG |
|  | PQ047625 | ACATATCATTCAAGTTTCTGACCTATCAGCTTTAGACGGTAGGGTATTGGCCTACCGTGGCTATGACGGGTAACGGGGAATTAGGGTTCGATTCCGGAGAGGGAGCCTGAGAAACGGCTACCACATCTAAGGAAGGCAGCAGGCGCGCAAATTACCCAATCCTAATACAGGGAGGTAGTGACAAGAAATAACAATACAGAACTTTACGGTTTTGTAATTGGAATGAGTTAAGTATAAACCCCTTAACAAGTATCAATTGGAGGGCAAGTCTGGTGCCAGCAGCCGCGGTAATTCCAGCTCCAATAGCGTATATTAAAGTTGTTGCAGTTAAAAAGCTCGTAGTTGGATTTCTGCTAATTTTTGCATACAATACTACGGTATTTATGTAAAATTAGCATAATCCGCATTACCTCGCGTATGCGGAATTTTACTTTGAGAAAATTAGAGTGCTTAAAGCAGGCAATTGCCTTGAATACTCCAGCATGGAATAATATTAAGGATTTTTATCCTTTTTATTGGTTCTAGGATAAAAATAATGATTAATAGGGACAGTTGGGGGCATTTGTATTTAACAGTCAGAGGTGAAATTCTTAGATTTGTTAAAGACAAACTACTGCGAAAGCATTTGCCAAGGATGTTTTCATTAATCAAGAACGAAAGTTAGGGGATCGAAGACGATCAGATACCGTCGTAGTCTTAACCATAAACTATGCCGACTAGAGATTGAGGTG |
|  | PQ047626 | TCAAGTTTCTGACCTATCAGCTTTAGACGGTAGGGTATTGGCCTACCGTGGCTATGACGGGTAACGGGGAATTAGGGTTCGATTCCGGAGAGGGAGCCTGAGAAACGGCTACCACATCTAAGGAAGGCAGCAGGCGCGCAAATTACCCAATCCTGACACAGGGAGGTAGGGACAAGAAATAACAATACAGGGCCTAACGGTCTTGTAATTGGAATGAGTGAAGTATAAACCCCTTTACGAGTATCAATTGGAGGGCAAGTCTGGTGCCAGCAGCCGCGGTAATTCCAGCTCCAATAGCGTATATTAAAGTTGTTGCAGTTAAAAAGCTCGTAGTTGGATTTCTGTTGTACATTATAATACTACTAAGGTAATATTATAATGGCAACATCCTTCCCACTATATTTTTTAATATATGGGAAATTTTACTTTGAGAAAATTAGAGTGCTTAAAGCAGGCAACTGCCTTGAATACTCCAGCATGGAATAATAAGTAAGGACTTTTGTCTTTCTTGTTGGTTCTAGGATAAAAGTAATGGTTAATAGGGACAGTTGGGGGCATTCGTATTTAACAGCCAGAGGTGAAATTCTTAGATTTGTTAAAGACGAACTACTGCGAAAGCATTTGCCAAGGATGTTTTCATTAATCAAGAACGAAAGTTAGGGGATCGAAGACGATCAGATACCGTCGTAGTCTTAACCATAAACTATGCCGACTAGAATTGAGT |
|  | PQ047627 | TACGTGACATATCATTCAAGTTTCTGACCTATCAGCTTTAGACGGTAGGGTATTGGCCTACCGTGGCTATGACGGGTAACGGGGAATTAGGGTTCGATTCCGGAGAGGGAGCCTGAGAAACGGCTACCACATCTAAGGAAGGCAGCAGGCGCGCAAATTACCCAATCCTAATACAGGGAGGTAGTGACAAGAAATAACAATACAGAACTTTACGGTTTTGTAATTGGAATGAGTTAAGTATAAACCCCTTAACAAGTATCAATTGGAGGGCAAGTCTGGTGCCAGCAGCCGCGGTAATTCCAGCTCCAATAGCGTATATTAAAGTTGTTGCAGTTAAAAAGCTCGTAGTTGGATTTCTGCTAATTTTTGCATACAATACCACGGTATTTATGTAAAATTAGCATAATCCGCATTACCTTGCGTATGCGGAATTTTACTTTGAGAAAATTAGAGTGCTTAAAGCAGGCAATTGCCTTGAATACTCCAGCATGGAATAATATTAAGGATTTTTATCCTTTTTATTGGTTCTAGGATAAAAATAATGATTAATAGGGACAGTTGGGGGCATTTGTATTTAACAGTCAGAGGTGAAATTCTTAGATTTGTTAAAGACAAACTACTGCGAAAGCATTTGCCAAGGATGTTTTCATTAATCAAGAACGAAAGTTAGGGGATCGAAGACGATCAGATACCGTCGTAGTCTTAACCATAAACTATGCCGACTAGAGATTGGAGGTG |
|  | PV124875 | TAGGGTTCGATTCCGGAGAGGGAGCCTGAGAAACGGCTACCACATCTAAGGAAGGCAGCAGGCGCGCAAATTACCCAATCCTGACACAGGGAGGTAGTGACAAGAAATAACAATACAGGGCCTAACGGTCTTGTAATTGGAATGAGTGAAGTATAAACCCCTTTACGAGTATCAATTGGAGGGCAAGTCTGGTGCCAGCAGCCGCGGTAATTCCAGCTCCAATAGCGTATATTAAAGTTGTTGCAGTTAAAAAGCTCGTAGTTGGATTTCTGTTGTACATTATAATACTACTAAGGTAATATTATAATGGCAACATCCTTCCCACTATATTTTTTAATATATGGGAAATTTTACTTTGAGAAAATTAGAGTGCTTAAAGCAGGCAACTGCCTTGAATACTCCAGCATGGAATAATAAGTAAGGACTTTTGTCTTTCTTGTTGGTTCTAGGATAAAAGTAATGGTTAATAGGGACAGTTGGGGGCATTCGTATTTAACAGCCAGAGGTGAAATTCTTAGATTTGTTAAAGACGAACTACTGCGAAAGCATTTGCCAAGGATGTTTTCATTAATCAAGAACGAAAGTTAGGGGATCGAAGACGATCAGATACCGTCGTAGTCTTAACCATAAACTATGCCGACTAGAGATTGGAGGTG |
|  | PV124876 | AGAAACGGCTACCACATCTAAGGAAGGCAGCAGGCGCGCAAATTACCCAATCCTGACACAGGGAGGTAGTGACAAGAAATAACAATACAGGGCCTAACGGTCTTGTAATTGGAATGAGTTAAGTATAAACCCCTTTACAAGTAGCAATTGGAGGGCAAGTCTGGTGCCAGCAGCCGCGGTAATTCCAGCTCCAATAGCGTATATTAAAGTTGTTGCAGTTAAAAAGCTCGTAGTTGGATTTCTGTTAATACTTATATACAATACCACGGTATTTATATAACATTAACATAATTCACATTACTTATTTAAAGTATGTGAAACTTTACTTTGAGAAAATTAGAGTGCTTAAAGCAGGCTATTGCCTTGAATACTCCAGCATGGAATAATATTAAAGATTTTTATCTTTCTTATTGGTTCTAGGATAAAAATAATGATTAATAGGGACAGTTGGGGGCATTTGTATTTAACAGTCAGAGGTGAAATTCTTAGATTTGTTAAAGACAAACTACTGCGAAAGCATTTGCCAAGGATGTTTTCATTAATCAAGAACGAAAGTTAGGGGATCGAAGACGATCAGATACCGTCGTAGTCTTAACCATAAACTATGCCGACTAGAGATTGGA |
|  | PV124877 | TGAGAAACGGCTACCACATCTAAGGAAGGCAGCAGGCGCGCAAATTACCCAATCCTAATACAGGGAGGTAGTGACAAGAAATAACAATACAGAACTTTACGGGTTTGGAATTGGAATGAGTTAAGTATAAACCCCTTAACAAGTATCAATTGGAGGGCAAGTCTGGGGCCAGCAGCCGCGGTAATTCCAGCTCCAATAGCGTATATTAAAGTTGTTGCAGTTAAAAAGCTCGTAGATGGATTTCTGCTAATTTTTACATACAATACTACGGTATTTATGTAAAATTAGCATAATCCGCATTACCTCGCGTATGCGGAACTTTACTTTGAGAAAATTAGAGTGCTTAAAGCAGGCAATTGCCTTGAATACTCCAGCATGGAATAATATTAAGGATTTTTATCCTTTTTATTGGTTCTAGGATAAAAATAATGATTAATAGGGACAGTTGGGGGCATTTGTATTTAACAGTCAGAGGTGAAATTCTTAGATTTGTTAAAGACAAACTACTGCGAAAGCATTTGCCAAGGATGTTTTCATTAATCAAGAACGAAAGTTAGGGGATCGAAGACGATCAGATACCGTCGTAGTCTTAACCATAAACTATGCCGACTAGAGATTGGAGGTTGTC |
|  | PV124878 | GGGAGCCTGAGAAACGGCTACCACATCTAAGGAAGGCAGCAGGCGCGCAAATTACCCAATCCTAATACAGGGAGGTAGTGACAAGAAATAACAATACAGAACCTTACGGTTTTGTAATTGGAATGAGTTAAGTATAAACCCCTTAACAAGTATCAATTGGAGGGCAAGTCTGGTGCCAGCAGCCGCGGTAATTCCAGCTCCAATAGCGTATATTAAAGTTGTTGCAGTTAAAAAGCTCGTAGTTGGATTTCTGTTAATTTTTATATATAATACTACGGTATTTATATAATATTAACATAATTCGCATTACTTTATGTATGCGAAATTTTACTTTGAGAAAATTAGAGTGCTTAAAGCAGGCAATTGCCTTGAATACTCCAGCATGGAATAATATTAAGGATTTTTATCCTTCTTATTGGTTCTAGGATAAAAATAATGATTAATAGGGACAGTTGGGGGCATTTGTATTTAACAGTCAGAGGTGAAATTCTTAGATTTGTTAAAGACAAACTACTGCGAAAGCATTTGCCAAGGATGTTTTCATTAATCAAGAACGAAAGTTAGGGGATCGAAGACGATCAGATACCGTCGTAGTCTTAACCATAAACTATGCCGACTAGAGATTGGAGGTTGTCCTTTACTCCTT |

Supplemental Figure S1 Pathogens detected by TAC. The *Cryptosporidium* qPCR assay on TAC targeted 18S rRNA gene, which demonstrated good sensitivity and specificity in the studies on human gastroenteritis. However, amplicon sequencing confirmed that a portion of the detection was due to algae that were likely present in the fecal samples of birds and possessed sequence similarity to *Cryptosporidium*. Therefore, conventional nested PCR followed by sequencing was performed on qPCR positives to confirm the *Cryptosporidium* detection.


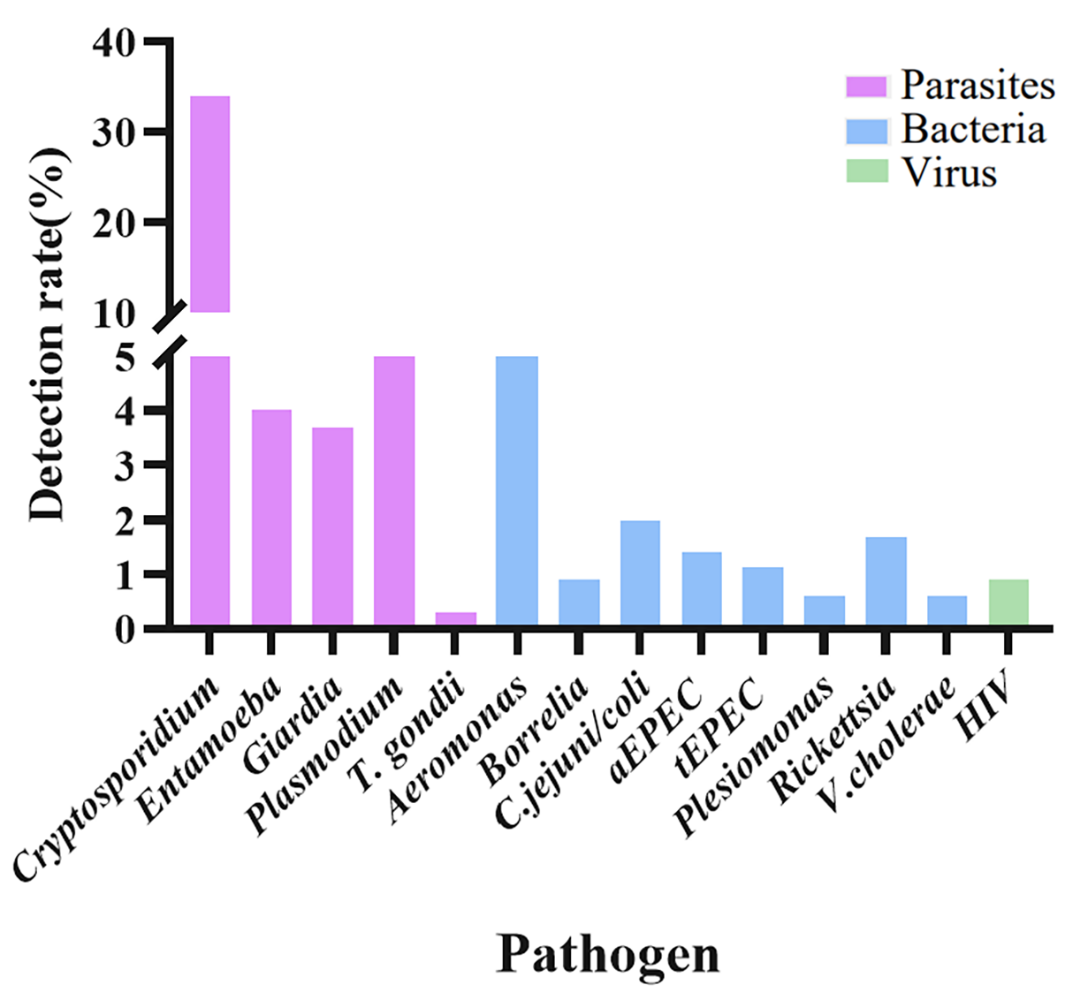


Supplemental Figure S2 Comparison of pathogen detection in *Anseriformes* and *Charadriiformes*. (A) Pathogen detection rate in *Anseriformes* and *Charadriiformes*, (B) the number of pathogens detected in *Anseriformes* and *Charadriiformes*, respectively.

A.


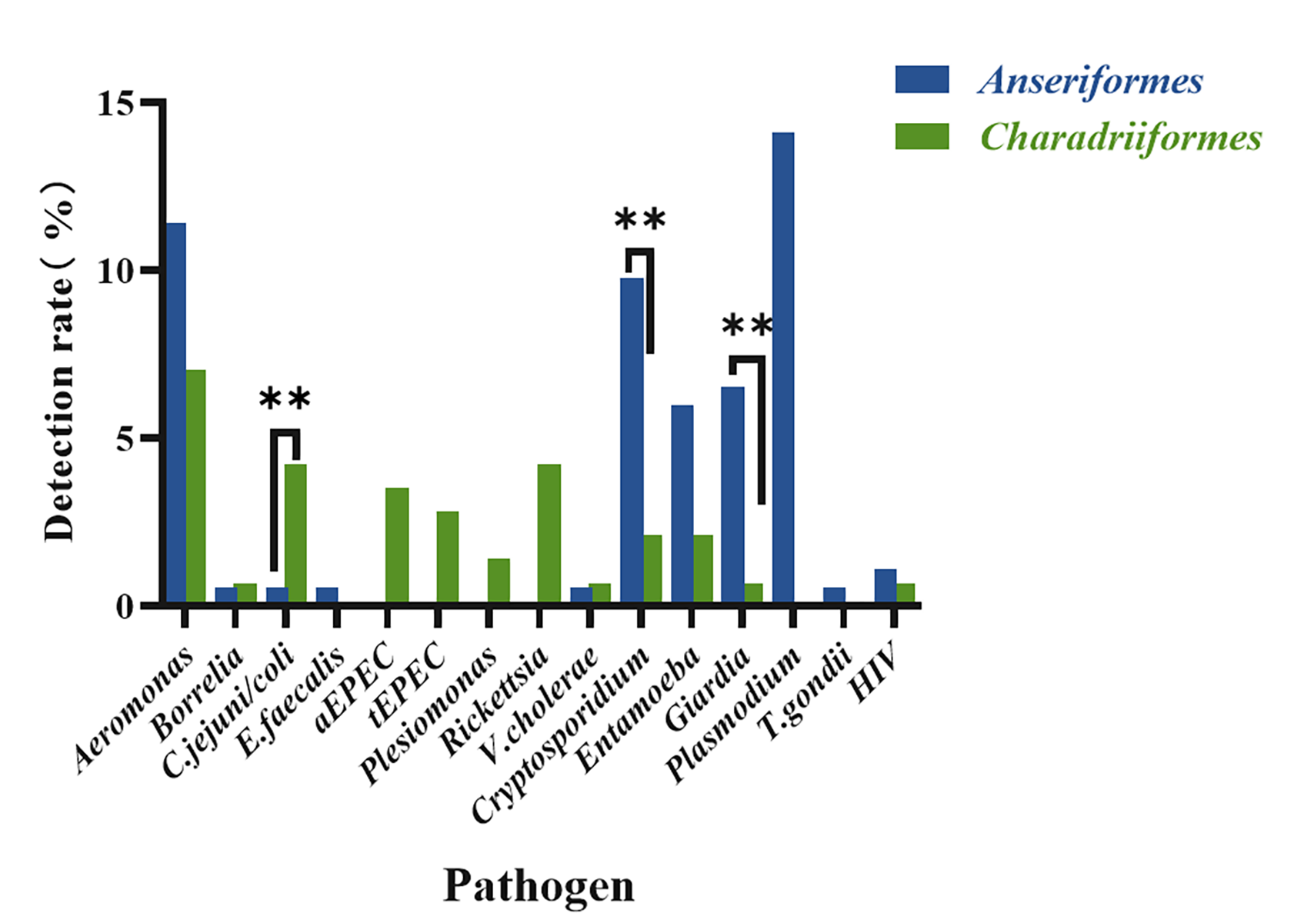


B


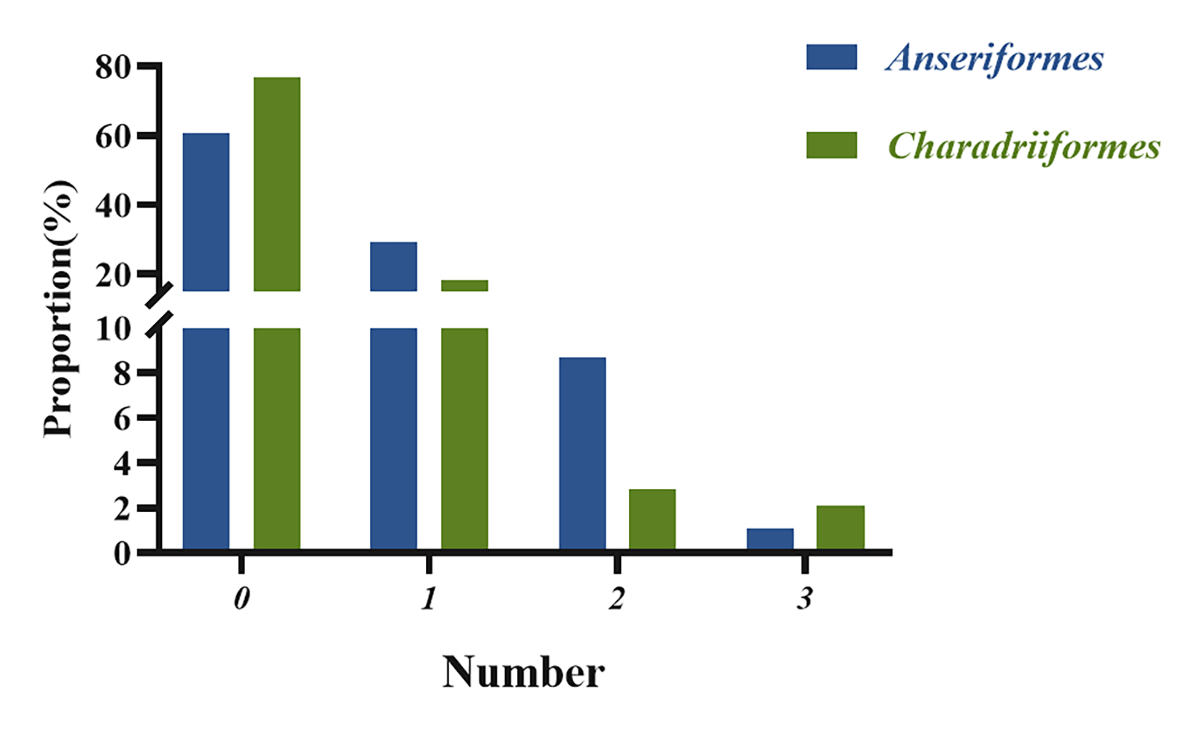


References

1. Łopińska A, Indykiewicz P, Skiebe E, et al (2020) Low Occurrence of Acinetobacter baumannii in Gulls and Songbirds. Pol J Microbiol 69:1–6. https://doi.org/10.33073/pjm-2020-011

2. Narciso AC, Martins WMBS, Almeida LGP, et al (2020) Healthcare-associated carbapenem-resistant OXA-72-producing Acinetobacter baumannii of the clonal complex CC79 colonizing migratory and captive aquatic birds in a Brazilian Zoo. Sci Total Environ 726:138232. https://doi.org/10.1016/j.scitotenv.2020.138232

3. Marks F, Liu J, Soura AB, et al (2021) Pathogens That Cause Acute Febrile Illness Among Children and Adolescents in Burkina Faso, Madagascar, and Sudan. Clin Infect Dis 73:1338–1345. https://doi.org/10.1093/cid/ciab289

4. Liang B, Ji X, Jiang B, et al (2022) Virulence, Antibiotic Resistance, and Phylogenetic Relationships of Aeromonas spp. Carried by Migratory Birds in China. Microorganisms 11:7. https://doi.org/10.3390/microorganisms11010007

5. Cardoso MD, Lemos LS, Roges EM, et al (2018) A comprehensive survey of Aeromonas sp. and Vibrio sp. in seabirds from southeastern Brazil: outcomes for public health. J Appl Microbiol 124:1283–1293. https://doi.org/10.1111/jam.13705

6. Liu J, Gratz J, Amour C, et al (2016) Optimization of Quantitative PCR Methods for Enteropathogen Detection. PLoS One 11:e0158199. https://doi.org/10.1371/journal.pone.0158199

7. Saggese MD, Noseda RP, Uhart MM, et al (2007) First detection of Bacillus anthracis in feces of free-ranging raptors from central Argentina. J Wildl Dis 43:136–141. https://doi.org/10.7589/0090-3558-43.1.136

8. Wielinga PR, Hamidjaja RA, Agren J, et al (2011) A multiplex real-time PCR for identifying and differentiating B. anthracis virulent types. Int J Food Microbiol 145 Suppl 1:S137-144. https://doi.org/10.1016/j.ijfoodmicro.2010.07.039

9. Cao H, Liu MC-J, Tong M-K, et al (2022) Comprehensive investigation of antibiotic resistance gene content in cfiA-harboring Bacteroides fragilis isolates of human and animal origins by whole genome sequencing. Int J Med Microbiol 312:151559. https://doi.org/10.1016/j.ijmm.2022.151559

10. Sacchi ABV, André MR, Calchi AC, et al (2021) Molecular and serological detection of arthropod-borne pathogens in carnivorous birds from Brazil. Vet Parasitol Reg Stud Reports 23:100539. https://doi.org/10.1016/j.vprsr.2021.100539

11. Molin Y, Lindeborg M, Nyström F, et al (2011) Migratory birds, ticks, and Bartonella. Infect Ecol Epidemiol 1:. https://doi.org/10.3402/iee.v1i0.5997

12. Liu J, Ochieng C, Wiersma S, et al (2016) Development of a TaqMan Array Card for Acute-Febrile-Illness Outbreak Investigation and Surveillance of Emerging Pathogens, Including Ebola Virus. J Clin Microbiol 54:49–58. https://doi.org/10.1128/JCM.02257-15

13. Kodani M, Yang G, Conklin LM, et al (2011) Application of TaqMan low-density arrays for simultaneous detection of multiple respiratory pathogens. J Clin Microbiol 49:2175–2182. https://doi.org/10.1128/JCM.02270-10

14. Liu J, Brennhofer SA, Zhang J, et al (2025) Effect of biannual azithromycin on respiratory pathogens among symptomatic children: results from the randomised Macrolides Oraux pour Réduire les Décès avec un Oeil sur la Résistance (MORDOR) I trial. BMJ Glob Health 10:e016043. https://doi.org/10.1136/bmjgh-2024-016043

15. Cicuttin GL, De Salvo MN, Venzal JM, Nava S (2019) Borrelia spp. in ticks and birds from a protected urban area in Buenos Aires city, Argentina. Ticks Tick Borne Dis 10:101282. https://doi.org/10.1016/j.ttbdis.2019.101282

16. Peñataro Yori P, Paredes Olórtegui M, Schiaffino F, et al (2023) Etiology of acute febrile illness in the peruvian amazon as determined by modular formatted quantitative PCR: a protocol for RIVERA, a health facility-based case-control study. BMC Public Health 23:674. https://doi.org/10.1186/s12889-023-15619-6

17. Najdenski H, Dimova T, Zaharieva MM, et al (2018) Migratory birds along the Mediterranean - Black Sea Flyway as carriers of zoonotic pathogens. Can J Microbiol 64:915–924. https://doi.org/10.1139/cjm-2017-0763

18. Hampton V, Kaestli M, Mayo M, et al (2011) Melioidosis in Birds and Burkholderia pseudomallei Dispersal, Australia. Emerg Infect Dis 17:1310–1312. https://doi.org/10.3201/eid1707.100707

19. Campbell S, Taylor B, Menouhos D, et al (2024) Performance of MALDI-TOF MS, real-time PCR, antigen detection, and automated biochemical testing for the identification of Burkholderia pseudomallei. J Clin Microbiol 62:e0096124. https://doi.org/10.1128/jcm.00961-24

20. Wu S, Jia R, Wang Y, et al (2024) Prevalence, Diversity, and Virulence of Campylobacter Carried by Migratory Birds at Four Major Habitats in China. Pathogens 13:230. https://doi.org/10.3390/pathogens13030230

21. Andrzejewska M, Grudlewska-Buda K, Śpica D, et al (2022) Genetic relatedness, virulence, and drug susceptibility of Campylobacter isolated from water and wild birds. Front Cell Infect Microbiol 12:1005085. https://doi.org/10.3389/fcimb.2022.1005085

22. Kasimov V, Dong Y, Shao R, et al (2022) Emerging and well-characterized chlamydial infections detected in a wide range of wild Australian birds. Transbound Emerg Dis 69:e3154–e3170. https://doi.org/10.1111/tbed.14457

23. Wolff BJ, Morrison SS, Winchell JM (2018) Development of a multiplex TaqMan real-time PCR assay for the detection of Chlamydia psittaci and Chlamydia pneumoniae in human clinical specimens. Diagn Microbiol Infect Dis 90:167–170. https://doi.org/10.1016/j.diagmicrobio.2017.11.014

24. Zlender T, Golob Z, Rupnik M (2022) Low Clostridioides difficile positivity rate in wild animal shelter in Slovenia. Anaerobe 77:102643. https://doi.org/10.1016/j.anaerobe.2022.102643

25. Ebani VV, Guardone L, Bertelloni F, et al (2021) Survey on the Presence of Bacterial and Parasitic Zoonotic Agents in the Feces of Wild Birds. Vet Sci 8:171. https://doi.org/10.3390/vetsci8090171

26. Kwit R, Zając M, Śmiałowska-Węglińska A, et al (2023) Prevalence of Enterococcus spp. and the Whole-Genome Characteristics of Enterococcus faecium and Enterococcus faecalis Strains Isolated from Free-Living Birds in Poland. Pathogens 12:836. https://doi.org/10.3390/pathogens12060836

27. Mduma E, Halidou T, Kaboré B, et al (2022) Etiology of severe invasive infections in young infants in rural settings in sub-Saharan Africa. PLoS One 17:e0264322. https://doi.org/10.1371/journal.pone.0264322

28. Sanches LA, Gomes M da S, Teixeira RHF, et al (2017) Captive wild birds as reservoirs of enteropathogenic E. coli (EPEC) and Shiga-toxin producing E. coli (STEC). Braz J Microbiol 48:760–763. https://doi.org/10.1016/j.bjm.2017.03.003

29. Liu Q, Bai X, Yang X, et al (2022) Identification and Genomic Characterization of Escherichia albertii in Migratory Birds from Poyang Lake, China. Pathogens 12:9. https://doi.org/10.3390/pathogens12010009

30. Lindsey RL, Garcia-Toledo L, Fasulo D, et al (2017) Multiplex polymerase chain reaction for identification of Escherichia coli, Escherichia albertii and Escherichia fergusonii. J Microbiol Methods 140:1–4. https://doi.org/10.1016/j.mimet.2017.06.005

31. Ahmed HA, Awad NFS, Abd El-Hamid MI, et al (2021) Pet birds as potential reservoirs of virulent and antibiotic resistant zoonotic bacteria. Comp Immunol Microbiol Infect Dis 75:101606. https://doi.org/10.1016/j.cimid.2020.101606

32. Kodani M, Yang G, Conklin LM, et al (2011) Application of TaqMan low-density arrays for simultaneous detection of multiple respiratory pathogens. J Clin Microbiol 49:2175–2182. https://doi.org/10.1128/JCM.02270-10

33. Mduma E, Halidou T, Kaboré B, et al (2022) Etiology of severe invasive infections in young infants in rural settings in sub-Saharan Africa. PLoS One 17:e0264322. https://doi.org/10.1371/journal.pone.0264322

34. Hubálek Z (2004) An annotated checklist of pathogenic microorganisms associated with migratory birds. J Wildl Dis 40:639–659. https://doi.org/10.7589/0090-3558-40.4.639

35. Gronesova P, Ficova M, Mizakova A, et al (2008) Prevalence of avian influenza viruses, Borrelia garinii, Mycobacterium avium, and Mycobacterium avium subsp. paratuberculosis in waterfowl and terrestrial birds in Slovakia, 2006. Avian Pathology 37:537–543. https://doi.org/10.1080/03079450802356953

36. Ghodbane R, Drancourt M (2013) Non-human sources of Mycobacterium tuberculosis. Tuberculosis (Edinb) 93:589–595. https://doi.org/10.1016/j.tube.2013.09.005

37. Kinzelman J, McLellan SL, Amick A, et al (2008) Identification of human enteric pathogens in gull feces at Southwestern Lake Michigan bathing beaches. Can J Microbiol 54:1006–1015. https://doi.org/10.1139/W08-096

38. Zhao H, Sun R, Yu P, Alvarez PJJ (2020) High levels of antibiotic resistance genes and opportunistic pathogenic bacteria indicators in urban wild bird feces. Environ Pollut 266:115200. https://doi.org/10.1016/j.envpol.2020.115200

39. Bassini-Silva R, de Castro Jacinavicius F, Muñoz-Leal S, et al (2023) Bacterial pathogens’ screening in Brazilian chigger mites (Trombidiformes: Trombiculidae), with the first report of ’Candidatus Rickettsia colombianensi’-like in avian-associated chiggers. Arch Microbiol 205:51. https://doi.org/10.1007/s00203-022-03393-9

40. Kwon H-J, Seong W-J, Kim J-H (2013) Molecular prophage typing of avian pathogenic Escherichia coli. Vet Microbiol 162:785–792. https://doi.org/10.1016/j.vetmic.2012.10.005

41. Kakulphimp J, Finch LR, Robertson JA (1991) Genome sizes of mammalian and avian Ureaplasmas. Int J Syst Bacteriol 41:326–327. https://doi.org/10.1099/00207713-41-2-326

42. Laviad-Shitrit S, Izhaki I, Halpern M (2019) Accumulating evidence suggests that some waterbird species are potential vectors of Vibrio cholerae. PLoS Pathog 15:e1007814. https://doi.org/10.1371/journal.ppat.1007814

43. Ayala AJ, Ogbunugafor CB (2023) When Vibrios Take Flight: A Meta-Analysis of Pathogenic Vibrio Species in Wild and Domestic Birds. Adv Exp Med Biol 1404:295–336. https://doi.org/10.1007/978-3-031-22997-8_15

44. Odyniec M, Stenzel T, Ławreszuk D, Bancerz-Kisiel A (2020) Bioserotypes, Virulence Markers, and Antimicrobial Susceptibility of Yersinia enterocolitica Strains Isolated from Free-Living Birds. Biomed Res Int 2020:8936591. https://doi.org/10.1155/2020/8936591

45. Talazadeh F, Ghorbanpoor M, Shahriyari A (2022) Candidiasis in Birds (Galliformes, Anseriformes, Psittaciformes, Passeriformes, and Columbiformes): A Focus on Antifungal Susceptibility Pattern of Candida albicans and Non-albicans Isolates in Avian Clinical Specimens. Topics in Companion Animal Medicine 46:100598. https://doi.org/10.1016/j.tcam.2021.100598

46. Raso TF, Werther K, Miranda ET, Mendes-Giannini MJS (2004) Cryptococcosis outbreak in psittacine birds in Brazil. Med Mycol 42:355–362. https://doi.org/10.1080/13693780410001712061

47. Widmer G, Köster PC, Carmena D (2020) *Cryptosporidium hominis* infections in non-human animal species: revisiting the concept of host specificity. International Journal for Parasitology 50:253–262. https://doi.org/10.1016/j.ijpara.2020.01.005

48. Myint T, Leedy N, Villacorta Cari E, Wheat LJ (2020) HIV-Associated Histoplasmosis: Current Perspectives. HIV AIDS (Auckl) 12:113–125. https://doi.org/10.2147/HIV.S185631

49. Holsback L, Cardoso MJL, Fagnani R, Patelli THC (2013) Natural infection by endoparasites among free-living wild animals. Rev Bras Parasitol Vet 22:302–306. https://doi.org/10.1590/S1984-29612013005000018

50. Abe N, Wu Z, Yoshikawa H (2003) Molecular characterization of Blastocystis isolates from birds by PCR with diagnostic primers and restriction fragment length polymorphism analysis of the small subunit ribosomal RNA gene. Parasitol Res 89:393–396. https://doi.org/10.1007/s00436-002-0782-5

51. Wang Y, Zhang K, Chen Y, et al (2021) Cryptosporidium and cryptosporidiosis in wild birds: A One Health perspective. Parasitol Res 120:3035–3044. https://doi.org/10.1007/s00436-021-07289-3

52. Totton SC, O’Connor AM, Naganathan T, et al (2021) A review of Cyclospora cayetanensis in animals. Zoonoses and Public Health 68:861–867. https://doi.org/10.1111/zph.12872

53. Kamel AA, Abdel-Latef GK (2021) Prevalence of intestinal parasites with molecular detection and identification of Giardia duodenalis in fecal samples of mammals, birds and zookeepers at Beni-Suef Zoo, Egypt. J Parasit Dis 45:695–705. https://doi.org/10.1007/s12639-020-01341-2

54. Cano L, de Lucio A, Bailo B, et al (2016) Identification and genotyping of Giardia spp. and Cryptosporidium spp. isolates in aquatic birds in the Salburua wetlands, Álava, Northern Spain. Vet Parasitol 221:144–148. https://doi.org/10.1016/j.vetpar.2016.03.026

55. al-Sallami S (1991) A possible role of crows in the spread of diarrhoeal diseases in Aden. J Egypt Public Health Assoc 66:441–449

56. Köller T, Hahn A, Altangerel E, et al (2020) Comparison of commercial and in-house real-time PCR platforms for 15 parasites and microsporidia in human stool samples without a gold standard. Acta Trop 207:105516. https://doi.org/10.1016/j.actatropica.2020.105516

57. Huang X, Chen Z, Yang G, et al (2022) Assemblages of Plasmodium and Related Parasites in Birds with Different Migration Statuses. Int J Mol Sci 23:10277. https://doi.org/10.3390/ijms231810277

58. Skírnisson K, Aldhoun JA, Kolárová L (2009) A review on swimmer’s itch and the occurrence of bird schistosomes in Iceland. J Helminthol 83:165–171. https://doi.org/10.1017/S0022149X09336408

59. Dubey JP, Murata FHA, Cerqueira-Cézar CK, et al (2021) Epidemiologic significance of Toxoplasma gondii infections in turkeys, ducks, ratites and other wild birds: 2009-2020. Parasitology 148:1–30. https://doi.org/10.1017/S0031182020001961

60. Joshua RA, Herbert WJ, White RG (1978) Acquisition by Trypanosoma brucei brucei of potential infectivity for man by passage through birds. Lancet 1:724–725. https://doi.org/10.1016/s0140-6736(78)90847-4

61. Martínez-Hernández F, Oria-Martínez B, Rendón-Franco E, et al (2022) Trypanosoma cruzi, beyond the dogma of non-infection in birds. Infect Genet Evol 99:105239. https://doi.org/10.1016/j.meegid.2022.105239

62. Li PH, Zheng PP, Zhang TF, et al (2017) Fowl adenovirus serotype 4: Epidemiology, pathogenesis, diagnostic detection, and vaccine strategies. Poult Sci 96:2630–2640. https://doi.org/10.3382/ps/pex087

63. Niczyporuk JS, Kozdrun W, Czekaj H, Stys-Fijol N (2021) Fowl adenovirus strains 1/A and 11/D isolated from birds with reovirus infection. PLoS One 16:e0256137. https://doi.org/10.1371/journal.pone.0256137

64. Shan T, Yang S, Wang H, et al (2022) Virome in the cloaca of wild and breeding birds revealed a diversity of significant viruses. Microbiome 10:60. https://doi.org/10.1186/s40168-022-01246-7

65. Abdelrahman Z, Li M, Wang X (2020) Comparative Review of SARS-CoV-2, SARS-CoV, MERS-CoV, and Influenza A Respiratory Viruses. Front Immunol 11:552909. https://doi.org/10.3389/fimmu.2020.552909

66. Tian Y, Yu T, Wang J, et al (2024) Genetic characterization of the first Deltacoronavirus from wild birds around Qinghai Lake. Front Microbiol 15:1423367. https://doi.org/10.3389/fmicb.2024.1423367

67. Dare RK, Fry AM, Chittaganpitch M, et al (2007) Human coronavirus infections in rural Thailand: a comprehensive study using real-time reverse-transcription polymerase chain reaction assays. J Infect Dis 196:1321–1328. https://doi.org/10.1086/521308

68. Lu X, Whitaker B, Sakthivel SKK, et al (2014) Real-time reverse transcription-PCR assay panel for Middle East respiratory syndrome coronavirus. J Clin Microbiol 52:67–75. https://doi.org/10.1128/JCM.02533-13

69. Leblebicioglu H, Eroglu C, Erciyas-Yavuz K, et al (2014) Role of migratory birds in spreading Crimean-Congo hemorrhagic fever, Turkey. Emerg Infect Dis 20:1331–1334. https://doi.org/10.3201/eid2008.131547

70. Gwee SXW, St John AL, Gray GC, Pang J (2021) Animals as potential reservoirs for dengue transmission: A systematic review. One Health 12:100216. https://doi.org/10.1016/j.onehlt.2021.100216

71. Spahr C, Knauf-Witzens T, Vahlenkamp T, et al (2018) Hepatitis E virus and related viruses in wild, domestic and zoo animals: A review. Zoonoses Public Health 65:11–29. https://doi.org/10.1111/zph.12405

72. Zhang X, Bilic I, Troxler S, Hess M (2017) Evidence of genotypes 1 and 3 of avian hepatitis E virus in wild birds. Virus Res 228:75–78. https://doi.org/10.1016/j.virusres.2016.11.028

73. Jesse ST, Ludlow M, Osterhaus ADME (2022) Zoonotic Origins of Human Metapneumovirus: A Journey from Birds to Humans. Viruses 14:677. https://doi.org/10.3390/v14040677

74. Bi Y, Yang J, Wang L, et al (2024) Ecology and evolution of avian influenza viruses. Curr Biol 34:R716–R721. https://doi.org/10.1016/j.cub.2024.05.053

75. Kolman JM, Folk C, Hudec K, Reddy GN (1976) Serologic examination of birds from the area of southern Moravia for the presence of antibodies against arboviruses of the groups Alfa, Flavo, Uukuniemi, Turlock and Bunyamwera supergroup. II. Wild living birds. Folia Parasitol (Praha) 23:251–255

76. Pankovics P, Boros Á, Mátics R, et al (2017) Ljungan/Sebokele-like picornavirus in birds of prey, common kestrel (Falco tinnunculus) and red-footed falcon (F. vespertinus). Infect Genet Evol 55:14–19. https://doi.org/10.1016/j.meegid.2017.08.024

77. de Barros B de CV, Chagas EN, Bezerra LW, et al (2018) Rotavirus A in wild and domestic animals from areas with environmental degradation in the Brazilian Amazon. PLoS One 13:e0209005. https://doi.org/10.1371/journal.pone.0209005

78. Ahlers LRH, Goodman AG (2018) The Immune Responses of the Animal Hosts of West Nile Virus: A Comparison of Insects, Birds, and Mammals. Front Cell Infect Microbiol 8:96. https://doi.org/10.3389/fcimb.2018.00096

79. Hoffman T, Lindeborg M, Barboutis C, et al (2018) Alkhurma Hemorrhagic Fever Virus RNA in Hyalomma rufipes Ticks Infesting Migratory Birds, Europe and Asia Minor. Emerg Infect Dis 24:879–882. https://doi.org/10.3201/eid2405.171369

80. Cao J, Hu Y, Liu F, et al (2020) Metagenomic analysis reveals the microbiome and resistome in migratory birds. Microbiome 8:26. https://doi.org/10.1186/s40168-019-0781-8
